# Lifestyle intervention Tai Chi for adult patients with type 2 diabetes mellitus: a PRIO-harms based overview of 17 systematic reviews

**DOI:** 10.3389/fendo.2023.1208202

**Published:** 2024-01-17

**Authors:** Furong Zhang, Xixi Chen, Xicen Liu, Xiaoyu Shen, Tianyu Liu, Fang Zeng, Rongjiang Jin

**Affiliations:** ^1^ College of Health Preservation and Rehabilitation, Chengdu University of Traditional Chinese Medicine, Chengdu, China; ^2^ Rehabilitation Department, Chengdu Second People’s Hospital, Chengdu, China; ^3^ Rehabilitation Department, Nuclear Industry 416 Hospital, Chengdu, China; ^4^ College of Sports and Health, Chengdu University of Traditional Chinese Medicine, Chengdu, China; ^5^ Acupuncture-Brain Science Research Center, Chengdu University of Traditional Chinese Medicine, Chengdu, China

**Keywords:** Tai Chi, type 2 diabetes, overview, AMSTAR2, PRIO-harms checklist, GRADE

## Abstract

**Objective:**

To systematically summarize current evidence and determine the clinical effectiveness and safety of Tai Chi for type 2 diabetes mellitus (T2DM) in adults by conducting an overview of systematic reviews (SRs).

**Methods:**

A systematic search encompassing five electronic databases was conducted until July 30, 2023, to identify relevant systematic reviews (SRs) based on randomized controlled trials (RCTs) concerning Tai Chi for T2DM. The methodological quality of the included SRs was assessed using the A MeaSurement Tool to Assess systematic Reviews (AMSTAR 2) and the Risk of Bias in Systematic Reviews (ROBIS) tool. The Preferred Reporting Items for Overview of Systematic Review (PRIO-harms) checklist was used to promote a more balanced reporting of benefits and harms in this overview. Corrected covered area (CCA) was used to calculate the degree of overlapping primary studies. Primary outcome measures were glycosylated hemoglobin (HbA1c) and fasting blood glucose (FBG), while secondary outcomes encompassed health-related quality measures. The GRADE (Grades of Recommendations, Assessment, Development, and Evaluation) framework was utilized to assess the quality of evidence for the outcome measures.

**Results:**

A total of 17 eligible SRs were included in this overview. One SR reported negative conclusions, while the remaining 16 reported positive ones on different outcomes. A total of 4 SRs reported adverse events, either absent or minor. Most of the SRs exhibited critically low quality (15/17) and a high risk of bias (14/17), as indicated by AMSTAR2 and ROBIS, respectively. The CCA was 12.14%, indicating a high degree of primary study overlapping. Evidence from 135 results for 24 outcomes concerning Tai Chi for T2DM was evaluated using the GRADE approach, most of which were rated very low.

**Conclusion:**

Tai Chi shows promise as a potentially effective and safe lifestyle intervention for adults with T2DM, particularly in improving HbA1c, FBG, BMI, and overall quality of life (QoL). However, these results should be cautiously interpreted due to methodological flaws observed in the current SRs and the low quality of the SRs based on GRADE. Furthermore, there is a compelling need for additional well-designed, high-quality RCTs and SRs to establish robust and conclusive evidence regarding the efficacy of Tai Chi for managing T2DM in the future.

**Systematic Review Registration:**

https://www.crd.york.ac.uk/PROSPERO/, identifier CRD 42019140988.

## 1 Introduction

Diabetes mellitus is a chronic non-communicable disease of global significance, impacting 463 million adults aged 20-79 years, which accounts for approximately 9.3% of the world’s population of this age range (1). Among these cases, more than 90% are of Type 2 Diabetes Mellitus (T2DM) (1). According to the IDF Diabetes Atlas (10th edition) (1), the prevalence of T2DM is projected to escalate to 12.2% by 2045, affecting 783 million people worldwide. The global health expenditure amounted to USD 966 billion in 2021. A nationwide population-based cross-sectional study reported that China’s diabetes incidence stands at 11.2%, encompassing a population exceeding 100 million individuals with diabetes (2). Managing this health-threatening and financially challenging issue has become a major public concern worldwide.

A sedentary lifestyle and physical inactivity are significant contributing factors to the development of T2DM. Within the spectrum of therapeutic approaches, exercise-based lifestyle interventions are gaining increasing prominence in addressing various chronic conditions, including T2DM (3). This is particularly evident in the comprehensive management strategy for T2DM, where exercise therapy is accorded comparable importance alongside blood glucose monitoring and pharmacological therapy. Tai Chi, a holistic mind-body practice blending mindfulness and physical movement, has emerged as a noteworthy clinical intervention. Recent research has suggested its potential as a cognitive treatment for older adults dealing with both T2DM and mild cognitive impairment (4). Moreover, Tai Chi’s efficacy extends to conditions like metabolic syndrome (5), fibromyalgia (6), knee osteoarthritis (7), Parkinson’s disease (8), and insomnia after breast cancer (9), as substantiated by clinical validation. In a bibliometric analysis (10), diabetes ranks 4th among the top 10 conditions for which Tai Chi offers health-enhancing benefits. However, akin to any exercise regimen, Tai Chi is not devoid of potential adverse effects, such as falls, bruises, and even fractures due to sporting injuries.

As such, it is imperative to consider the safety implications of Tai Chi alongside investigating its manifold advantages.

Several systematic reviews (SRs) have investigated the efficacy and safety of Tai Chi as a lifestyle intervention for T2DM. Some of these SRs propose that, compared to other aerobic exercises, Tai Chi could better improve fasting blood glucose (FBG), glycated hemoglobin (HbA1c), balance, and quality of life (QoL) and reduce the high-density lipoprotein (HDL) and body mass index (BMI) of T2DM patients (11–13). Conversely, a different perspective has been presented by several other SRs (14–16), leading to unfavorable conclusions. A consensus among outcome measures remains elusive, and the quality of reporting and methodology in SRs themselves can significantly impact confidence in the derived evidence. As a promising and cost-effective exercise intervention for T2DM management, its effectiveness needs further validation. Hence, this overview of SRs was conducted and reported under the guidance of the checklist of Preferred Reporting Items for Overview of Systematic Reviews (PRIO-harms) (17), aiming to critically appraise the effectiveness, safety, and methodological rigor of currently published SRs based on RCTs that explore Tai Chi’s potential for adult patients with T2DM.

## 2 Method

### 2.1 Protocol and registration

The study protocol was registered on the PROSPRO platform (https://www.crd.york.ac.uk/PROSPERO/), with an assigned register: CRD 42019140988. The protocol was published in advance (18).

### 2.2 Literature search

A systematic search was conducted across five electronic databases: Web of Science, Cochrane Database of Systematic Reviews, MEDLINE via Pubmed, EMBASE via Ovid), and one Chinese electronic database, China National Knowledge Infrastructure (CNKI) from the inception of each database until July 30, 2023, without any language restrictions. The search terms employed included but were not limited to the following: (systematic review OR meta-analysis) AND (tai chi OR taiji OR tai chi chuan OR taijiquan OR chi, tai OR jiquan, tai OR quan, taiji OR chuan, taichi) AND (type 2 diabetes OR type 2 diabetes mellitus OR diabetes mellitus, type 2 OR diabetes mellitus OR noninsulin-dependent OR non-insulin dependent), (系统评价 OR meta分析) AND (太极拳 OR 太极) AND (糖尿病OR 消渴OR 脾瘅), with necessary adjustments made to accommodate the diverse syntax requirements of the respective databases. The full search strategy is listed in Appendix A. In addition, study registries in PROSPERO and gray literature like conference articles and dissertations were searched for supplementary information.

### 2.3 Inclusion and exclusion criteria

The Population, Intervention, Comparison, Outcome, and Study design (PICOS) strategy was employed in establishing the inclusion and exclusion criteria.

(1) **Participants (P):** Participants in the primary studies were diagnosed with T2DM according to recognized clinical guidelines.

(2) **Intervention (I):** Tai Chi (with no restriction on style), or Tai Chi with usual care were eligible interventions in this study. Usual care includes standard anti-diabetic agents (metformin, acarbose, insulin, etc.), nursing, and health education. Exclusions encompassed Tai Chi variations involving specific apparatus-based exercises like Tai Chi sword, Tai Chi ball, and similar adaptations derived from Tai Chi.

(3) **Comparisons (C)**: The control interventions included usual care or treatment as usual and other exercise interventions apart from Tai Chi, such as walking, resistance training, yoga, dancing, etc., placebo (sham exercise), or waiting list. SRs comparing different styles of Tai Chi were excluded.

(4) **Outcome measures (O)**: Primary outcomes were HbA1c and FBG. Secondary outcomes included postprandial blood glucose (PBG), fasting serum insulin (FINS), index of homeostasis model assessment of insulin resistance (HOMA-IR), body mass index (BMI), the Short-Form Health Survey (SF-36), BMI, lipids-related indices (total cholesterol (TCh), triglycerides (TG), high-density lipoprotein (HDL), low-density lipoprotein (LDL)), cognition, balance, and emotion. Safety was measured by adverse events (AEs) such as falls, fainting, bruises, etc.

(5) **Study design (S)**: In this overview, only SRs that encompassed more than one RCT were included. Furthermore, SR protocols, duplicates, network SRs, or SRs lacking complete data or whose full text was unavailable were excluded.

### 2.4 Eligibility assessment and data extraction

All retrieved citations were imported into Endnote X9 software, and duplicates were filtered and removed by the software. Two independent reviewers (Z-FR and C-XX) screened all the titles and abstracts for relevance, followed by a meticulous assessment of full-text eligibility. A cross-verification process was executed to ensure consistency. The exclusion list with corresponding justifications was provided in Appendix B. A predefined template for data extraction was used to collect the following information from each eligible SR: study features (first author, year of publication, country and region, language and publication type, number of included RCTs and patients recruited), methodological features (participants, interventions, controls, outcomes, and RCT quality assessment tools), and main conclusions of SRs. In instances involving duplicated data, priority was accorded to the most recent iteration due to its heightened methodological rigor and encompassment of a larger number of RCTs. For any instances of missing or incomplete data, the corresponding author was contacted via email. Any disagreements were settled by introducing a third reviewer (J-RJ).

### 2.5 Assessment of methodological quality and risk of bias

A MeaSurement Tool to Assess systematic Reviews (AMSTAR 2) (19) was used to assess the methodological quality of included SRs. AMSTAR is a popular methodological quality assessment tool for RCT-based SRs (19). AMSTAR 2, an update of AMSTAR released in 2017, incorporates an expansion from 11 items to 16, with a focus on 7 critical domains (items 2, 4, 7, 9, 11, 13, and 15). This updated version accommodates the assessment of both randomized and non-randomized controlled trials, utilizing predefined key items to establish a quality rating. Risk of Bias in Systematic reviews (ROBIS) (20) was applied to evaluate the risk of bias of the included SRs. ROBIS operates through three distinct phases (1): assess relevance (optional) (2), identify concerns with the review process, and (3) judge the risk of bias. Phase 2 includes 4 domains by which bias may be involved in an SR: study eligibility criteria; identification and selection of studies; data collection and study appraisal; and synthesis and findings (20). The evaluation of all included systematic reviews was conducted independently by two reviewers (L-XC, S-XY) in accordance with previously outlined instructions (21). Likewise, in case of any discrepancies, a consensus was reached through group discussion.

### 2.6 Reporting quality and overlapping assessment

We employed the checklist with PRIO-harms (17) to promote a more balanced reporting of benefits and harms in this overview. Developed from the PRISMA, PRISMA harms, and PRISMA-P statements, and guidelines from related methodological review articles (17), the checklist has a set of 27 items (56 subitems) for reporting benefits and harms in overviews of healthcare interventions (see Appendix C). It is noteworthy that the PRIO-harms checklist encompasses a dedicated segment for addressing overlaps (instances where primary studies are encompassed within multiple eligible systematic reviews, potentially leading to a duplication of outcomes). To tackle this, we employed the concept of Corrected Covered Area (CCA) as a recommended approach for evaluating overlapping effects.

While overviews based on SRs provide evidence with a broader summary of the current information available, their statistical power can become disproportionate when primary studies (usually RCTs) are incorporated into multiple SRs (22). Therefore, in conjunction with the PRIO-harms checklist (17), the CCA (22) was used to quantify the extent of overlap in this overview. This overlap was further visualized using an upset plot. The calculation formula was as follows: CCA=(N-r)/(rc-r) (22), where N represents the number of included publications (RCTs in this study, including double counting); r stands for the number of included index publications (rows in the citation matrix) defined as the first appearance of a primary publication; c is the number of included SRs (columns in the citation matrix). The CCA values were categorized as follows: 0 to 5 for slight overlap, 6 to 10 for moderate overlap, 11 to 15 for high overlap, and exceeding 15 for very high overlap (22). The upset plot was made by the online tool Bioladder (https://www.bioladder.cn/web/#/chart/16).

### 2.7 Evidence quality assessment

The Grades of Recommendations, Assessment, Development, and Evaluation (GRADE) (23) framework was used to assess the evidence quality regarding the main outcomes of the meta-analysis. This assessment encompassed five key domains: study limitations (risk of bias), inconsistency, indirectness, imprecision, and publication bias. The evaluation process was conducted using the GRADEpro GDT online (http://www.guidelinedevelopment.org/) by two authors (Z-FR and C-XX) separately. Any discrepancies that arose were resolved through group discussions to achieve a consensus.

### 2.8 Data synthesis and presentation

The SRs included in the overview were descriptively analyzed. Characteristics of each SR including methodology quality (AMSTAR 2 and ROBIS), interventions, outcomes, and conclusions were summarized in tables and figures. The reporting quality of this overview was demonstrated in the PRIO-harms checklist (see Appendix C). The result of the overlapping assessment was displayed in an upset plot. The evidence profile and summary of findings were generated using the GRADEpro GDT online software.

### 2.9 Patient and public involvement

Given that this is a literature review, no patient participation was involved in this overview. Beyond the inclusion in peer-reviewed journals, the insights derived from the study findings are intended for dissemination across medical and nursing institutions and communities including patients affected by T2DM.

## 3 Results

### 3.1 Results of literature search and selection

**Figure 1:**
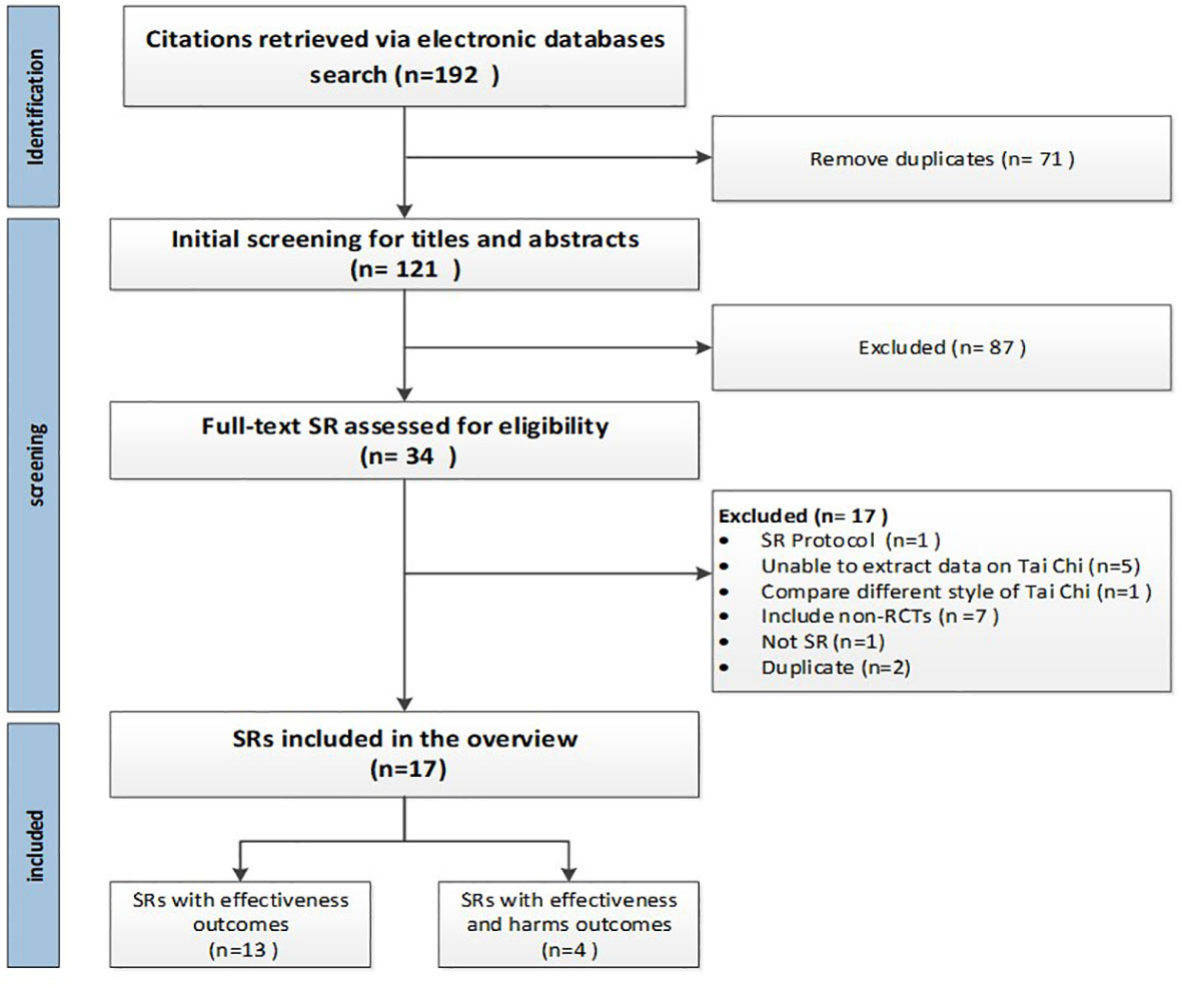
Flow diagram of literature search and study selection. The search yielded a total of 192 citations; 71 duplicates were removed and another 87 were excluded through initial screening of titles and abstracts. A total of 34 underwent full-text assessment, with 17 excluded, and finally, 17 SRs were included. The flowchart of the study selection is shown in **Figure 1**.

The search yielded a total of 192 citations. A total of 71 duplicates were removed and an additional 87 citations were excluded through the initial screening of titles and abstracts. A comprehensive full-text assessment was conducted on 34 citations, resulting in the exclusion of 17. Consequently, 17 SRs were ultimately included in this study. The flowchart of the study selection is shown in **Figure 1**. The exclusion list with explanations is shown in Appendix B.

### 3.2 Characteristics of included SRs

The basic characteristics of the included SRs are presented in **Table 1**. A total of 17 SRs (11, 14–16, 24–36) published between 2014 and 2022 were included in this overview. Among these, 10 SRs were reported in Chinese and the remaining 7 were in English. Dissertations accounted for 3 of the SRs (28, 31, 32) and the remaining 14 were all journal articles. The majority of these SRs (16 out of 17) were authored by researchers based in China, with the remaining one being from South Korea (16). The number of RCTs included in the SRs ranged from 4 to 24, encompassing patient cohorts ranging from 55 to 1314 individuals. The age of enrolled T2DM patients ranged from 35.6 to 79.2 years old, and the disease duration ranged from 0.4 to 23 years. Four tools were employed to assess the methodological quality of RCTs across the SRs. Specifically, the Cochrane Collaboration Risk of Bias tool was employed in 12 SRs, the (modified) Jadad scale in 3 SRs (15, 35, 36), the PEDro scale in 1 SR (14), and the key evaluation list of JBI RCT in 1 SR (30). Only 1 SR (25) used the GRADE system to assess the evidence quality and 6 SRs (11, 16, 25, 26, 29, 36) were reported under the guidance of the PRISMA Statement.

**Table 1:** Characteristics of included SRs.

First-author year country/language (article type)	No. of RCTs (patients)	Age/course of disease (years) (range)	Participants state	RCT tools	PRISMA Statement	Fundings	Synthesis	Interventions	Comparators	Duration	Outcomes	Main conclusions
XZ Wang 2022 (24) China/English (journal article)	19 (1220)	51.3-70.4/NA	T2DM	RoB	NA	National fund 2013-42	Meta	TC (Chen-style, Yang-style, simplified 24-style, Eight methods and five Steps)	regular exercise (with personal care, daily walking, or no exercise).	4-24 weeks	FBG, HbA1c, TCh, TG, HDL, LDL,	TC shows significant superiority in improving FBG, HbA1c, TG, and HDL-C in T2DM patients.
Cai 2022 (25) China/English (journal article)	24 (1314)	NA	T2DM or with complications	RoB	Y	University fund 90011451310034	Meta	TC (Chen-style, simplified 24-style, mixed Sun and Yang style other styles.)	usual care, other exercise (walking, balancing, social dance, calisthenics, and stretching)	8-24 weeks	FBG, HbA1c, IR, FIN, TCh, TG, HDL, LDL, BMI, SBP, DBP, WC, QoL, balance, anxiety, safety, and adverse events	Compared with usual care, TC may improve HbA1c (with clinical significance), FBG, FIN, BMI, DBP, and QoL in T2DM patients. The effects of TC were similar to those of other exercises on HbA1c, FBG, TC, TG, HDL, LDL, BMI, and WC.
Yin 2022 (26) China/Chinese (journal article)	14 (790)	NA	T2DM	RoB	Y	NA	Meta	TC	usual care, other aerobic exercise (walking, dancing, square dancing, etc.)	8 weeks-4 years	FBG, HbA1c, FINS, TCh, TG, BMI.	TC exercise helps T2DM patients to control blood sugar, improve insulin sensitivity, reduce body mass index, etc., yet has no effect on lipid levels.
Wang 2022 (10) China/English (journal article)	7 (905)	44.7-79.2/NA	T2DM	RoB	Y	Municipal funds PWZxk2017-10, GLRI2018-01.	Meta	TC	usual care	NA	glucose, ABC, single limb standing test	TC performed better than usual care in improving elderly T2DM patients’ glucose and QoL.
Guo 2021 (11) China/English (journal article)	23 (613)	46.8-70.4/NA	T2DM	RoB	Y	National fund 2019YFC1710301, Provincial fund 2018Y2002.	Meta	TC (21-style Yang and Sun, 24-style, simplified 24-style, 20-style Yang and Sun, 12-style Yang and Sun, etc.)	clinical conventional therapy, sham exercise, other aerobic exercise (brisk walking, dancing, square dancing, etc.)	NA	FBG, PBG, HbA1c, TCh, TG, HDL, LDL, BMI, SBP, DBP, HOMA-IR, FINS, WC, GSP.	TC outweighed CCT on improving metabolic control and body composition indices, while advantaged over aerobic exercise only in terms of improving HbA1c and HDL.
Ge 2020 (27) China/Chinese (journal article)	13 (856)	NA	T2DM	RoB	NA	NA	Meta	TC	usual treatment or blank control	NA	FBG, HbA1c; TCh, TG, HDL-C, LDL-C	TC regulated the blood glucose and lipoprotein of T2DM patients.
Xun 2019 (28) China/Chinese (Dissertation)	41 (272)	42-74.7/0.4-19	T2DM	RoB	NA	NA	Meta	TC (simplified 24-style, Dayuan style,12-style Yang and Sun, simplified 99-style, Yang, Lin, Chen, etc.)	other aerobic exercise, No intervention, usual treatment (social dancing, square dancing, walking, etc.).	4-24 weeks	FBG, HbAlc, FINS	TC had better effects on FBG, HbAlc, and FINS in T2DM patients.
Su 2019 (29) China/Chinese (journal article)	15 (1099)	I:60.7 ± 12.2, NA C:63.2 ± 8.6, NA	T2DM or with metabolic syndrome	RoB	PY	National fund 2015B053	Meta	TC (24-style, 20-styles Sun and Yang, 37-style Chen, 32- style Yang, etc.)	no intervention; diet control; health education; medication	8-24 weeks	FBG, HbA1c, HOMA, blood lipids	TC improved the glucose and insulin sensitivity of T2DM patients. Different styles of TC may be the main source of heterogeneity.
Zhou 2019 (14) China/English (journal article)	23 (1217)	35.6-69.5/1-23	T2DM	PEDro	NA	NA	Meta	TC (Chen, Sun, Yang, Lin, etc.)	No intervention; usual treatment or exercise, sham exercise	4-24 weeks	FBG, HbA1c, blood pressure, blood lipids BMI, balance, QoL	TC improved blood glucose and quality of life in T2DM patients but had no effect on balancing and fasting insulin.
Yu 2018 (30) China/English (journal article)	8 (446)	49.0-70.4 /1.4-15.0	T2DM	JBI Checklist	NA	NA	Meta	TC (24-style, 18-style Lin, Yang, Sun, etc.)	usual treatment; sham exercise	12-24 weeks	FBG, HbA1c, blood pressure, BMI, WC, SF-36	TC improved the FBG, BMI, and QoL of T2DM patients, yet the impact on blood pressure and waist circumference was uncertain (because the number of studies is too small)
Chao 2018 (15) China/English (journal article)	14 (798)	48.0–64.0/NA	T2DM	modified Jadad	NA	NA	Meta	TC (24-style, 24-style of Yang, Lin, Yang, etc.)	No exercise, other aerobic exercise group (ballroom dancing, qigong, ordinary aerobic exercise, etc.)	4-24 weeks	FBG, HbA1c, 2hPBG	TC effectively improved the patient’s blood glucose level and HbA1c. Long-term practice exerted better effects.
Q Wang 2017 (31) China/Chinese (Dissertation)	14 (785)	NA/2-23	T2DM or with hypertension, obesity	RoB	NA	NA	Meta	TC (simplified 24-style) + basic treatment	Other aerobic exercise, no exercise, health education, medication	8-24 weeks	FBG, HbA1c	TC significantly improved the FBG of T2DM patients compared with the blank control, yet there was no significant difference compared with the aerobic exercise group.
CY Wang 2017 (32) China/Chinese (Dissertation)	15 (830)	48.0-64.0/NA	T2DM	Jadad	NA	NA	Meta	TC or TC + medicine	medicine, other exercise (walking, ballroom dancing, random exercise, stretching, etc.)	4-24 weeks	FBG, HbA1c, 2hPBG, blood lipids, etc.	Compared with no exercise, TC effectively reduced FBG, HbA1c, and 2hPBG, yet there was no significant difference when compared with other exercises.
Tang 2017 (33) China/Chinese (journal article)	11 (764)	NA	T2DM	RoB	NA	National fund 15BSH124	Meta	TC (24-style, simplified 24-style, 20-style Yang and Sun, Chen, etc.)	regular exercise	8-24 weeks	FBG, HbA1c, BMI, blood lipids, SF-36	TC controlled blood glucose, reduced weight and blood lipids, and improved QoL.
Liu 2017 (34) China/Chinese (journal article)	10 (773)	NA	T2DM	RoB	NA	National fund 81494550	Meta	TC + medicine	random exercise, or no exercise	8-24 weeks	FBG, HbA1c, blood lipids, SF-36	TC adjusted the blood glucose and lipid metabolism of patients and improved QoL.
Zhang 2016 (35) China/Chinese (journal article)	4 (55)	NA	T2DM	modified Jadad	NA	NA	Meta	TC	NA	>12 weeks	blood glucose, whole blood and red blood cell insulin receptors R1 and R2	Long-term TC lowered the blood glucose and increased the bioactivity of insulin receptors R1 and R2. However, the quality of the included literature was low.
Lee 2014 (16) Korea/English (journal article)	15 (754)	NA/0.5-10.64	T2DM	RoB	Y	NA	Meta	TC (Young, Sun, Chen, etc.) or TC + other treatments	no exercise, usual treatment, waiting list	12-24 weeks	FBG, HbA1c, QoL	The existing evidence was insufficient to show that TC is effective for T2DM.

RCT, randomized controlled trial; NA, not available; I, intervention group; C, control group; T2DM, type 2 diabetes mellitus; Rob, the Cochrane risk of bias (ROB) tool; PEDro, the Physiotherapy Evidence Database (PEDro) scale; Jadad, Jadad Scale; TC, Tai Chi; ABC, activities-specific balance confidence; QoL, quality of life; SF-36, 36-Item Short Form Health Survey; FBG, fasting blood glucose; PBG, postprandial blood glucose; HbA1c, glycated hemoglobin; TCh, total cholesterol; TG, triglyceride; HDL &LDL, high-density lipoprotein & low-density lipoprotein; BMI, body mass index; SBP & DBP, systolic blood pressure & diastolic blood pressure; HOMA-IR, index of homeostasis model assessment of insulin resistance; FINS, fasting serum insulin; WC, waist circumference; GSP, glycated serum protein; PRISMA, the Preferred Reporting Items for Systematic Reviews and Meta-Analyses; Y, yes.

A total of 7 SRs (11, 24, 25, 29, 33, 34, 36) reported their funding sources, predominantly deriving support from national and provincial entities. All 17 SRs applied meta-analysis. The interventions included Tai Chi, either as a standalone intervention or in conjunction with usual care, while the control groups mainly received treatment as usual or usual care, sham exercises, no treatment or waiting list, and other exercises (walking, dancing, etc.). The duration of interventions varied between 4 weeks and 4 years. The main outcomes included indices related to glucose metabolism, insulin sensitivity, blood lipid profiles, and indicators of QoL. In terms of the results and conclusions, the SR published in 2014 (16) reported a negative conclusion that the existing evidence was insufficient to show that TC is effective for T2DM; while the remaining SRs consistently indicated favorable outcomes for Tai Chi, often demonstrating superior efficacy compared to control conditions, which encompassed usual care alone, waiting lists, and alternative aerobic exercises.

### 3.3 Methodological appraisal results of included SRs by AMSTAR 2

The findings of AMSTAR 2 methodological quality assessment are shown in **Table 2**. Among the 17 SRs under evaluation, 15 were rated critically low quality due to the identification of multiple critical weaknesses (items 2, 4, 7, 9, 11, 13, and 15). Additionally, 2 SRs (14, 25) were rated low quality. The majority of the included SRs excelled in implementing the PICO components, study selection, and data extraction in duplicate, followed by choosing appropriate methods for data pooling, assessing the risk of bias with proper tools, and providing appropriate explanations for the results. However, only 2 SRs (11, 25) reported study protocols. Moreover, none of the SRs explained the choice of RCT as the study design, provided a list of excluded studies with justifications, or disclosed funding information.

**Table 2:** Results of methodology assessment of SRs included by the AMSTAR 2.

SRs	Q1	Q2	Q3	Q4	Q5	Q6	Q7	Q8	Q9	Q10	Q11	Q12	Q13	Q14	Q15	Q16	Quality
XZ Wang 2022 (24)	Y	N	N	PY	Y	Y	N	PY	PY	N	Y	N	Y	Y	Y	Y	Critically low
Cai 2022 (25)	Y	Y	N	PY	Y	Y	N	PY	PY	N	Y	Y	Y	Y	Y	Y	**Low**
Yin 2022 (26)	Y	N	N	PY	Y	N	N	PY	PY	N	Y	N	Y	Y	N	N	Critically low
Wang 2022 (10)	Y	N	N	PY	N	Y	N	N	N	N	Y	N	N	N	Y	Y	Critically low
Guo 2021 (11)	Y	Y	N	PY	N	Y	N	N	Y	N	Y	Y	N	N	N	Y	Critically low
Ge 2020 (27)	Y	N	N	N	Y	Y	N	N	Y	N	Y	N	PY	N	PY	N	Critically low
Xun 2019 (28)	Y	N	N	N	Y	N	N	N	N	N	Y	Y	Y	Y	Y	N	Critically low
Su 2019 (29)	Y	N	N	PY	N	N	N	PY	Y	N	Y	Y	Y	N	N	N	Critically low
Zhou 2019 (14)	Y	N	N	PY	Y	Y	N	PY	PY	N	Y	Y	Y	Y	Y	Y	**Low**
Yu 2018 (30)	Y	N	N	PY	Y	Y	N	PY	PY	N	Y	Y	Y	Y	PY	N	Critically low
Chao 2018 (15)	Y	N	N	PY	Y	Y	N	PY	PY	N	N	Y	Y	Y	Y	Y	Critically low
Q Wang 2017 (31)	Y	N	N	PY	N	Y	N	PY	Y	N	Y	Y	Y	Y	Y	N	Critically low
CY Wang 2017 (32)	Y	N	N	PY	Y	Y	N	PY	Y	N	Y	Y	Y	Y	Y	N	Critically low
Tang 2017 (33)	Y	N	N	PY	Y	Y	N	PY	Y	N	Y	Y	Y	Y	N	N	Critically low
Liu 2017 (34)	Y	N	N	PY	Y	Y	N	PY	Y	N	Y	Y	Y	Y	Y	N	Critically low
Zhang 2016 (35)	Y	N	N	PY	N	N	N	PY	PY	N	Y	Y	N	N	Y	N	Critically low
Lee 2014 (16)	Y	N	N	PY	Y	Y	N	PY	Y	N	N	Y	Y	Y	N	Y	Critically low
**Total of Y**	17	2	0	0	12	13	0	0	8	0	15	13	13	12	10	7	

Y, yes; PY, partial yes; N, no. Numbers in bold are critical items.

AMSTAR 2 items:

1. Did the research questions and inclusion criteria for the review include the components of PICO?

**2.** Did the report of the review contain an explicit statement that the review methods were established prior to the conduct of the review and did the report justify any significant deviations from the protocol?

3. Did the review authors explain their selection of the study designs for inclusion in the review?

**4**. Did the review authors use a comprehensive literature search strategy?

5. Did the review authors perform study selection in duplicate?

6. Did the review authors perform data extraction in duplicate?

**7**. Did the review authors provide a list of excluded studies and justify the exclusions?

8. Did the review authors describe the included studies in adequate detail?

**9**. Did the review authors use a satisfactory technique for assessing the risk of bias (RoB) in individual studies that were included in the review?

10. Did the review authors report on the sources of funding for the studies included in the review?

**11**. If meta-analysis was performed did the review authors use appropriate methods for statistical combination of results?

12. If meta-analysis was performed, did the review authors assess the potential impact of RoB in individual studies on the results of the meta-analysis or other evidence synthesis?

**13**. Did the review authors account for RoB in individual studies when interpreting/discussing the results of the review?

14. Did the review authors provide a satisfactory explanation for, and discussion of, any heterogeneity observed in the results of the review?

**15**. If they performed quantitative synthesis, did the review authors carry out an adequate investigation of publication bias (small study bias) and discuss its likely impact on the results of the review?

16. Did the review authors report any potential sources of conflict of interest, including any funding they received for conducting the review?

### 3.4 Risk of bias appraisal results of included SRs

**Figure 2:**
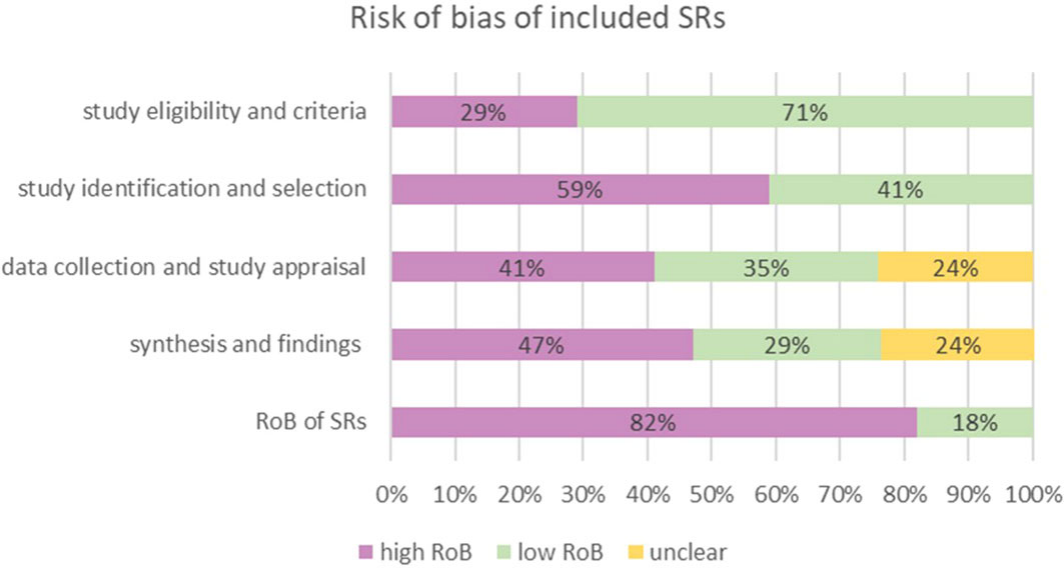
Results of risk bias of SRs included assessed by ROBIS. For Phase 2, four domains were investigated. In Domain 1 (study eligibility criteria), 12 SRs (71%) were rated low risk, 7 SRs (41%) were rated low risk in Domain 2 (identification and selection of studies), 6 (35%) were rated low risk in Domain 3 (collection and study appraisal), and 5 (29%) were rated low risk in Domain 4 (synthesis and findings). In Phase 3 (risk of bias in the review), 3 SRs (18%) were rated low risk.

Among the 17 SRs, 3 SRs (11, 14, 25, 30) were rated low risk of bias, and the remaining 14 were assigned a high risk of bias rating. The detailed results are shown in **Figure 2** and **Table 3**. In the assessment of bias risk using ROBIS, 16 SRs (94%) were rated low risk in Phase 1 (assessing relevance). For Phase 2, four domains were investigated. In Domain 1 (study eligibility criteria), 12 SRs (71%) were rated low risk, 7 SRs (41%) low risk in Domain 2 (identification and selection of studies), 6 (35%) low risk in Domain 3 (collection and study appraisal), and 5 (29%) low risk in Domain 4 (synthesis and findings). Three SRs (18%) were rated low risk in Phase 3 (risk of bias in the review).

**Table 3:** Results of risk bias of the included SRs assessed by ROBIS.

SRs	Phase 1	Phase 2				Phase 3
	Assessing relevance	Domain 1 Study eligibility criteria	Domain 2 Identification and selection of studies	Domain 3 Data collection and study appraisal	Domain 4 Synthesis and findings	RoB of SR
XZ Wang 2022 (24)	☺	☺	☺	?	?	☹
Cai 2022 (25)	☺	☺	☺	☺	☺	☺
Yin 2022 (26)	☺	☺	☺	?	?	☹
Wang 2022 (10)	☺	☺	☹	?	?	☹
Guo 2021 (11)	☺	☺	☺	☺	☹	☹
Ge 2020 (27)	☺	☺	☹	☹	☹	☹
Xun 2019 (28)	☺	☹	☹	☹	☹	☹
Su 2019 (29)	☺	☹	☹	☹	☹	☹
Zhou 2019 (14)	☺	☺	☺	☺	☺	☺
Yu 2018 (30)	☺	☺	☺	☺	☺	☺
Chao 2018 (15)	☺	☺	☹	☺	☹	☹
Q Wang 2017 (31)	☺	☺	☹	?	☺	☹
CY Wang 2017 (32)	☹	☹	☹	☹	☺	☹
Tang 2017 (33)	☺	☹	☹	☹	☹	☹
Liu 2017 (34)	☺	☺	☹	☺	☹	☹
Zhang 2016 (35)	☺	☹	☹	☹	☹	☹
Lee 2014 (16)	☺	☺	☺	☹	?	☹

☺, low risk bias; ☹, high risk bias; **?**, unclear risk bias; ROBIS, risk of bias in systematic reviews; RoB, risk of bias

### 3.5 Reporting quality results of this overview

The reporting quality of this overview was demonstrated in the PRIO-harms checklist (see Appendix C).

### 3.6 Effectiveness of Tai Chi for adult patients with T2DM

The effectiveness indices of Tai Chi for adult patients with T2DM are summarized in **Table 4**. Out of the 17 SRs, 16 (including 3 with low risk of bias) arrived at affirmative conclusions, while the earliest study by Lee in 2014 (16) reported a negative one.

**Table 4:** Effectiveness and safety indices of Tai Chi for T2DM.

Study	HbA1c1(%)	Outcomes							AEs	Risk of bias
		BG (mmol/L)	FINS	HOMA-IR	Blood lipids	BMI(kg/m^2^)	QoL	Balance		
XZ Wang 2022 (24)	☑ ^a^	☑FBG ^a^	NA	NA	☑TG ^a^	NA	NA	NA	NA	High
Cai 2022 (25)	☑ ^a^ ☒ ^b^	☑ ^b^ ☒ ^a^ FBG	☑ ^a^	☒ ^a^	☒ ^a,b^ TCh, TG, HDL, LDL	☑ ^a^☒ ^b^	☑ ^a^ physical functioning; mental and general health; social function; emotional role; and vitality.	NA	No	**Low**
Yin 2022 (26)	☑ ^c^	☑FBG ^c^	☑ ^c^	NA	☒ ^c^ TCh, TG,	☑ ^c^	NA	NA	NA	High
Wang 2022 (10)	NA	☑BG ^b^	NA	NA	NA	NA	NA	☑ ^b^ ABC, single limb standing test	NA	High
Guo 2021 (11)	☑ ^a,b^	☑ ^b^ FBG, PBG	☑ ^b^	☑ ^b^	☑ ^b^ TCh, TG, ☒ TCh ^a^,TG ^a^,HDL ^a,b^, LDL^b^	☑^b^	NA	NA	No	High
Ge 2020 (27)	☑ ^c^	☑FBG ^c^	NA	NA	☑^c^TG, HDL, LDL☒^c^TCh	NA	NA	NA	NA	High
Xun 2019 (28)	☑ ^b^	☑FBG ^b^	☒ ^b^	NA	NA	NA	NA	NA	NA	High
Su 2019 (29)	☑ ^c^	☑ ^c^ FBG, PBG	☒ ^c^	☑ ^c^	☒TCh ^c^	NA	NA	NA	NA	High
Zhou 2019 (14)	☑ ^c^	☑FBG ^c^	☑ ^c^	☑ ^c^	☑TCh ^c^	☑ ^c^	☑ ^c^ exercise, pain, social	☒ ^c^ single-leg stance	NA	**Low**
Yu 2018 (30)	☑ ^c^	☑FBG ^c^	NA	NA	NA	☑ ^c^	☑ ^c^ physical, mental domains	NA	NA	**Low**
Chao 2018 (15)	☑ ^d^ ☒ ^a^	☑ ^d^ ☒ ^a^ FBG, PBG	NA	NA	NA	NA	NA	NA	NA	High
Q Wang 2017 (31)	☑ ^c^	☑ FBG ^c^	NA	NA	NA	NA	NA	NA	No or very slight	High
CY Wang 2017 (32)	☑ ^d^☒ ^a^	☑ ^d^☒ ^a^ FBG, PBG	NA	NA	☑ ^d^TCh, LDL ☒ TCh^a^, TG^a,d^, HDL^a,d^, LDL^a^	NA	NA	NA	NA	High
Tang 2017 (33)	☑ ^a^	☑BG ^a^	NA	NA	☑ TG^a^ ☒TCh^a^	☑ ^c^	☑ ^a^ social, emotional, overall health	NA	NA	High
Liu 2017 (34)	☑ ^c^	☑FBG ^c^	NA	NA	☑^c^TG, HDL, LDL ☒TCh	NA	☑ ^c^ social, mental, overall health	NA	NA	High
Zhang 2016 (35)	NA	☑ BG ^c^	NA	NA	NA	NA	NA	NA	NA	High
Lee 2014 (16)	☒ ^a,b,e^	☑FBG ^b^ ☒FBG ^a^	NA	NA	NA	NA	☒ ^(not pooled)^	NA	No or very slight	High

NA, not available; ☑, Tai Chi is superior to controls; ☒, Tai Chi is inferior to controls; HbA1c, glycated hemoglobin; BG, blood glucose; FBG, fasting blood glucose; PBG, postprandial blood glucose; FINS, fasting serum insulin; HOMA-IR, index of homeostasis model assessment of insulin resistance; TCh, total cholesterol; TG, triglyceride; HDL &LDL, high-density lipoprotein & low-density lipoprotein; BMI, body mass index; QoL, quality of life; Aes, adverse events.

a , TC vs. other exercise

b , TC vs. conventional clinical treatment/usual care

c , TC vs. unspecified control (a, b, sham exercise, no intervention, health education, etc.)

d , nonexercised

e , no treatment or waiting list.

#### 3.6.1 Glycated hemoglobin A1c

HbA1c, a stable glycated hemoglobin molecule, serves as a reflective measure of average blood glucose levels over the past 2 to 3 months, and it assumes a pivotal role in both the management and diagnosis of diabetes mellitus (37, 38). The HbA1c results were presented in 15 SRs. Except for Lee’s 2014 study (16), the remaining 14 SRs (11, 14, 15, 24–34) concurred that Tai Chi demonstrated significant efficacy in reducing HbA1c levels among T2DM patients when compared to controls (mean difference [MD]: -1.48 to -0.59).

#### 3.6.2 Blood glucose indices

All 17 SRs reported the results of blood glucose indices and 14 SRs (11, 14–16, 24–36) reported inconsistent results on FBG outcome. While Tai Chi exhibited superior efficacy in reducing FBG when compared to usual care, no statistically significant differences were observed in comparison to other exercise interventions (15, 16). A total of 3 SRs (15, 29, 32) reported a lower 2hPBG and another 3 (33, 35, 36) demonstrated lower blood glucose levels in the Tai Chi group.

#### 3.6.3 Insulin resistance indices

Five SRs (11, 14, 25, 26, 28) reported lower FINS (mean difference [MD]: -3.00 to -0.28) levels in the Tai Chi group, of which, fouR (11, 14, 25, 29) reported HOMA-IR and one (25) reported a negative result.

#### 3.6.4 Blood lipids

Blood lipids indices mainly include TCh, TG, HDL, and LDL. A total of 10 SRs (11, 14, 24–27, 29, 32, 33, 35) reported results of blood lipids. With the exception of 2 SRs (25, 26), the remaining reviews uniformly indicated that Tai Chi yielded superior reductions in blood lipid indices compared to usual care or no treatment control. See Appendix D for details.

#### 3.6.5 BMI

BMI, calculated as an individual’s weight in kilograms divided by the square of their height in meters (kg/m²), serves as an established metric for gauging body fat. In the evaluation of BMI outcomes, six SRs (11, 14, 25, 26, 30, 33) reported results of BMI (MD:-1.64 to -0.39), reaching a consensus that Tai Chi could reduce BMI more effectively than usual care. Compared with other exercise, Tai Chi did not show an advantage in reducing BMI (25).

#### 3.6.6 Quality of life

Six SRs (14, 16, 25, 30, 33, 34) reported outcomes on QoL as evaluated by SF-36, diabetes-specific QoL (DSQoL) instruments. These evaluations encompassed domains such as physical functioning, physical role, pain, general health, vitality, social function, emotional role, and mental health. Lee’s study in 2014 (16) reported no difference between Tai Chi and the control group in promoting the QoL of T2DM patients. Conversely, the remaining five indicated Tai Chi’s capacity to augment QoL in aspects relating to physiology (physical functioning, bodily pain), psychology (mental/emotional health), sociality (social participation), and overall health. Yu’s investigation in 2018 (30) reported Tai Chi practice for at least 150 min per week could improve the mental domain of QoL (MD=6.54; 95%CI [0.77, 12.3]; I^2 =^ 61%, p = 0.03), indicating a dose-effect relation of Tai Chi for the QoL of T2DM patients.

#### 3.6.7 Balance

Two SRs (14, 36) reported outcomes on balance, evaluated by the activities-specific balance confidence (ABC), single-limb standing test with eyes open or closed, and single-leg stance assessments. Zhou’s study in 2019 (14) found no difference between Tai Chi and the control group in increasing the duration of single-leg stance (MD=2.71; 95% CI (–3.29, 8.71); p=0.376; I^2 ^= 63.8%, p=0.063). Conversely, Wang’s research in 2022 (36) demonstrated that their Tai Chi group had higher ABC scale scores (MD=9.26, 95%CI [6.68, 11.83], p < 0.001) and single limb standing test scores (MD=8.38, 95%CI [4.02, 12.74], p <0.001) than the controls.

### 3.7 Safety of Tai Chi for T2DM

The safety is mainly measured by the adverse events reported in the SRs, as evidenced in **Table 4**. Four SRs (11, 16, 25, 31) reported adverse events characterized as minimal or negligible adverse reactions or injuries, with no instances of severe adverse effects reported. Moreover, a 14-SRs-based overview reported that Tai Chi may be beneficial for improving balance and reducing falls in older people and patients with Parkinson’s disease (39). Consequently, the consensus within the available evidence suggests that Tai Chi remains a favorable safety intervention for individuals with T2DM.

### 3.8 Results of overlapping assessment by CCA

**Figure 3:**
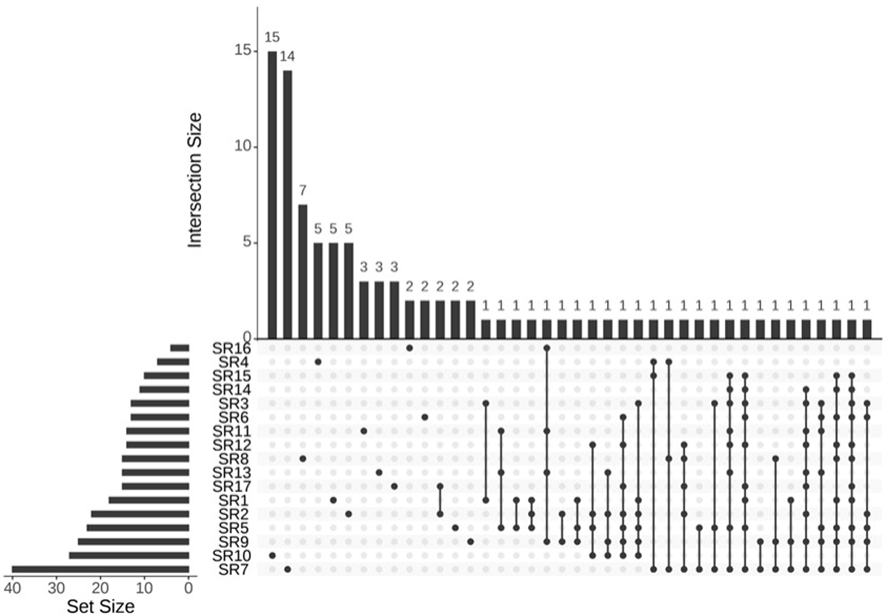
Upset plot of overlapping assessment. RCTs shared among all included SRs. Upset plot of the intersection of different RCTs across SRs. The horizontal bar graph (set size) on the left shows the number of RCTs included in each SR. The upper bar graph shows the number of RCTs for each overlapping combination. Black connected circles indicate which SRs are involved in each intersection.

The total number (N) of included publications was 292 (including double counting), the number of index publications (without double counting) r was 96, and the number of SRs c was 17. Based on the formula CCA=(N-r)/(rc-r)*100%, the CCA of this overview was 12.14%, indicating a high degree of overlap (22). The upset plot is shown in **Figure 3**.

### 3.9 Evidence quality based on GRADE framework

The GRADE evidence profile and GRADE summary of the findings of the included SRs are displayed in **Supplementary Table 1** and **Table 5**, respectively. Evidence from 135 results concerning 24 outcomes of Tai Chi for adults wit`h T2DM was summarized. The quality assessment indicated that all the evidence was appraised as being of low or very low quality. Among the five contributing factors leading to downgrading, the foremost consideration was limitation (risk of bias), followed by impression (not reaching the optimal information size), inconsistency (o great heterogeneity), and potential publication bias.

**Table 5:** GRADE summary of findings of included SRs.

Outcomes	Relative effect (95% CI); heterogeneity	No. of RCTs (participants)	Evidence certainty (GRADE)	Notes and comments	SRs
**HbA1c1**	MD -1.10[-1.78, -0.24]; I^2^ = 98%	16 (991)	Very low	RCTs with high RoB and high heterogeneity	XZ Wang 2022 (24)
	MD -0.80 [-1.05, -0.54]; I^2^ = 18.3% TC vs. CCT MD -0.15 [-0.33, 0.04]; I^2^ = 6.2% TC vs. AE	6 (282) 5 (401)	Very low Low	RCTs with high RoB, imprecision, and publication bias RCTs with high RoB and imprecision.	Cai 2022 (25)
	MD -0.81[-1.31,-0.32]; I^2^ = 85%	8 (499)	Very low	RCTs with high RoB, high heterogeneity, small sample size, and publication bias	Yin 2022 (26)
	MD -0.73[-1.03, 0.43]; I^2^ = 82% TC vs. CCT	9 (749)	Very low	RCTs with high RoB, high heterogeneity, small sample size, and publication bias	Guo 2021 (11)
	MD -0.33[-0.61, 0.04]; I^2^ = 48% TC vs. AE	5 (504)	Very low	RCTs with high RoB, moderate heterogeneity, small sample size, and publication bias	
	SMD -0.585(-0.784,−0.386); I^2^ = 95.2%	NA	Very low	RCTs with high RoB, high heterogeneity, unclear sample size, and publication bias	Ge 2020 (27)
	SMD -0.50(-0.60, -0.40); I^2^ = 74.4%	24 (774)	Very low	RCTs with high RoB, high heterogeneity, small sample size, and publication bias	Xun 2019 (28)
	MD -0.76 [-1.38, -0.14]; I^2^ = 97%	7 (515)	Very low	RCTs with high RoB, high heterogeneity, small sample size, publication bias	Su 2019 (29)
	WMD -0.53(-0.62%,-0.44%); I^2^ = 43.4%	12 (714)	Very Low	RCTs with high RoB, moderate heterogeneity, small sample size, and publication bias	Zhou 2019 (14)
	MD -1.25[-−2.53, 0.03]; I^2^ = 99%	5 (294)	Very low	RCTs with high RoB, high heterogeneity, very small sample size, and publication bias	Yu 2018 (30)
	MD -0.73[-0.95, -0.52]; I^2^ = 57% MD -0.19[-0.37, 0.00]; I2 = 65%	7 (293) 7 (372)	Very Low Very Low	RCTs with high RoB, moderate heterogeneity, very small sample size, and publication bias	Chao 2018 (15)
	SMD -0.68[-0.86, -0.51]; I^2^ = 88%	8 (583)	Very Low	RCTs with high RoB, high heterogeneity, very small sample size, and publication bias	Q Wang 2017 (31)
	MD-0.98[-1.65, -0.31]; I^2^ = 97% TC vs. nonexercised MD -0.25 [-0.52, 0.01]; I^2^ = 68% TC vs. AE	8 (356) 5 (303)	Very Low Very Low	RCTs with high RoB, moderate to high heterogeneity, very small sample size, and publication bias.	CY Wang 2017 (32)
	MD -0.77 (-1.16, -0.39); I^2^ = 93%	7 (572)	Very Low	RCTs with high RoB, high heterogeneity, small sample size, and publication bias	Tang 2017 (33)
	MD -0.59 (-0.73, -0.44); I^2^ = 45%	7 (645)	Very Low	RCTs with high RoB, moderate heterogeneity, small sample size, and publication bias	Liu 2017 (34)
	MD 0.00[-0.31, 0.31]; I^2^ = 0% TC vs. AE	2 (148)	Very Low	RCTs with high RoB, very small sample size, and publication bias	Lee 2014 (16)
	MD -0.54[-1.23, 0.15]; I^2^ = 14% TC vs. drug MD -1.58[-3.83, 0.67]; I^2^ = 95% TC vs. no treatment	3 (127) 2 (84)	Very Low Very Low	RCTs with high RoB, low heterogeneity, very small sample size, and publication bias RCTs with high RoB, high heterogeneity, small sample size, and publication bias	
**FBG**	MD-0.79[-1.12,-0.64]; I^2^ = 75%	18 (1069)	Very Low	RCTs with high RoB, high heterogeneity, and publication bias	XZ Wang 2022 (24)
	SMD -0.58 [-0.86, -0.31]; I^2^ = 53.2% TC vs. CCT MD -0.99 [-0.49,0.32]; I^2^ = 6.2% TC vs. AE	11 (533) 4 (363)	Low Very Low	RCTs with high RoB and moderate heterogeneity RCTs with high RoB, inconsistency, and publication bias	Cai2022 (27)
	MD -0.85[-1.22,-0.38]; I^2^ = 75% TC vs. CCT MD 0.14[-0.32, 0.59]; I^2^ = 39% TC vs. AE	8 (385) 9 (344)	Very Low Very Low	RCTs with high RoB, high heterogeneity, and publication bias	Yin2022 (26)
	SMD -0.62[-0.85, 0.40]; I^2^ = 63%, TC vs. CCT SMD -0.03[-0.30, 0.23]; I^2^ = 60%, TC vs. AE	15 (1023) 8 (619)	Very Low Very Low	RCTs with high RoB, moderate heterogeneity, and publication bias RCTs with high RoB, moderate heterogeneity, small sample size, and publication bias	Guo 2021 (11)
	SMD -0.67(-0.87, -0.47); I2 = 53.2%	21 (1115)	Very Low	RCTs with high RoB, moderate heterogeneity, and publication bias	Zhou2019 (14)
	SMD -0.38(-0.46, -0.29); I^2^ = 42.3%	39 (1044)	Very Low	RCTs with high RoB, moderate heterogeneity, and publication bias	Xun 2019 (28)
	SMD -0.85 [-1.17, -0.52]; I^2^ = 83%	14 (2230)	Very Low	RCTs with high RoB, high heterogeneity, and publication bias	Su2019 (29)
	MD -1.14[-1.78, -0.50]; I^2^ = 67%	6 (303)	Very Low	RCTs with high RoB, moderate heterogeneity, small sample size, and publication bias	Yu 2018 (30)
	MD-1.39[-1.95, -0.84];I^2^ = 86%	10 (489)	Very Low	RCTs with high RoB, moderate heterogeneity, small sample size, and publication bias	Chao 2018 (15)
	SMD -0.24[-0.39, -0.09]; I^2^ = 38.3%	12 (708)	Very Low	Included RCTs with high RoB, moderate heterogeneity, small sample size, and publication bias	Q Wang 2017 (31)
	MD -1.11 [-1.65, -0.56]; I^2^ = 79% TC vs. nonexercised MD -0.32 [-0.80, 0.17]; I^2^ = 64% TC vs. AE	9 (480) 5 (285)	Very Low Very Low	RCTs with high RoB, moderate to high heterogeneity, small sample size, and publication bias	CY Wang 2017 (32)
	MD -0.39 (-0.54, -0.24); I^2^ = 47%	9 (727)	Very Low	RCTs with high RoB, moderate heterogeneity, small sample size, and publication bias	Liu 2017 (34)
	MD -0.03[-0.49, 0.42];I^2^ = 39% TC vs. AE	4 (212)	Very Low	RCTs with high RoB, moderate heterogeneity, very small sample size, and publication bias	Lee 2014 (16)
	MD -1.57[-2.34, -0.80];I^2^ = 0% TC vs. drug	4 (188)	Very Low	RCTs with high RoB, very small sample size, and publication bias	
**2hPBG**	MD -1.03 [-1.34, -0.73]; I^2^ = 0%	2 (140)	Very Low	RCTs with high RoB, very small sample size, and publication bias	Su 2019 (29)
	MD -2.07 [-2.89, -1.26]; I^2^ = 0%	5 (162)	Very Low	RCTs with high RoB, very small sample size, and publication bias	Chao 2018 (15)
	MD -2.05 [-3.12, -0.09]; I^2^ = 0% TC vs. nonexercised MD -0.67 [-2.17, 0.83]; NA. TC vs. AE	3 (105) 1 (27)	Very Low Very Low	RCTs with high RoB, very small sample size, and publication bias	CY Wang 2017 (32)
**Glucose**	MD -12.47 [-21.20,-3.73]; I^2^ = 32%	5 (275)	Very Low	RCTs with high RoB, very small sample size, and publication bias	Wang 2022 (10)
	MD -0.74 (-1.32, -0.16); I^2^ = 70%	7 (354)	Very Low	RCTs with high RoB, moderate heterogeneity, very small sample size, and publication bias	Tang 2017 (33)
	MD -0.43[-0.03, -0.84]; I^2^ = 0%	4 (118)	Very Low	RCTs with high RoB, very small sample size, and publication bias.	Zhang 2016 (35)
**FINS**	SMD-0.68[-1.16,-0.20]; I^2^ = 65.1%	6 (224)	Low	RCTs with high RoB, imprecision, and publication bias	Cai 2022 (25)
	MD-3.00[-4.04,-1.97]; I^2^ = 67%	6 (280)	Very Low	Included RCTs with high RoB, high heterogeneity, and suspicion of publication bias	Yin 2022 (26)
	MD -2.63 (-4.51, -0.76); I^2^ = 68%	7 (500)	Very Low	RCTs with high RoB, moderate heterogeneity, small sample size, and publication bias	Guo 2021 (11)
	MD -0.28 (-0.44, -0.12); I^2^ = 34.5%	12 (308)	Very Low	RCTs with high RoB, low heterogeneity, very small sample size, and publication bias	Xun 2019 (28)
	SMD -0.32 (-0.71,-0.07); I^2^ = 73.3%	8 (500)	Very Low	RCTs with high RoB, high heterogeneity, small sample size, and publication bias	Zhou 2019 (14)
**HOMA-IR**	MD-0.60 [11.30, 0.10]; I^2^ = 0	3 (75)	Very Low	RCTs with high RoB, imprecision, and publication bias	Cai 2022 (25)
	MD -1.02 (-1.39, -0.64); I^2^ = 0%	3 (255)	Low	RCTs with high RoB, low heterogeneity, very small sample size, and publication bias.	Guo 2021 (11)
	MD -0.69[-0.06, -1.31]; I^2^ = 59%	4 (377)	Very Low	RCTs with high RoB, moderate heterogeneity, very small sample size, and publication bias	Su 2019 (29)
	WMD -0.41 (-0.78, -0.04); I^2^ = 0%	7 (332)	Very Low	RCTs with high RoB, low heterogeneity, very small sample size, and publication bias.	Zhou 2019 (14)
**TCh**	MD-0.27[-0.60,0.05]; I^2^ = 82%	10 (662)	Very Low	RCTs with high RoB, high heterogeneity, small sample size, and publication bias	XZ Wang 2022 (24)
	SMD -0.44[-0.94,0.06], I^2^ = 80.9% TC vs. CCT MD -0.27[-0.95,0.42]; I^2^ = 89.3% TC vs. AE	6 (382) 3 (262)	Very Low Very Low	RCTs with high RoB and imprecision. RCTs with high RoB, inconsistency, and imprecision;	Cai 2022 (25)
	MD -0.11[-0.32,0.09]; I^2^ = 46%	10 (499)	Very Low	RCTs with high RoB, moderate heterogeneity, small sample size, and publication bias	Yin2022 (26)
	SMD -0.51[-0.88, -0.14]; I^2^ = 84%TC vs. CCT	11 (868)	Very low	RCTs with high RoB, high heterogeneity, and publication bias	Guo 2021 (11)
	MD -0.08[-0.24, 0.09]; I^2^ = 36% TC vs. AE	5 (423)	Very low	RCTs with high RoB, small sample size, and suspicion of publication bias	
	SMD -0.418(-0.897,0.061); I^2^ = 84.9%	7 (NA)	Very low	RCTs with high RoB, high heterogeneity, unclear sample size, and publication bias	Ge 2020 (27)
	SMD -0.67[-1.38, 0.03]; I^2^ = 92%	5 (1238)	Very low	RCTs with high RoB, high heterogeneity, and publication bias	Su 2019 (29)
	SMD-0.59[-0.90,-0.27]; I^2^ = 66.6%	9 (658)	Very Low	Included RCTs with high RoB, moderate heterogeneity, small sample size, and publication bias	Zhou 2019 (14)
	MD -0.45, [-0.75, -0.14]; I^2^ = 83% TC vs. nonexercised MD -0.04, [-0.33, 0.25]; I^2^ = 68% TC vs. AE	4 (215) 4 (258)	Very Low Very Low	RCTs with high RoB, high heterogeneity, very small sample size, and publication bias.	CY Wang 2017 (32)
	MD -0.08 [-0.33, 0.48]; I^2^ = 80%	6 (518)	Very Low	RCTs with high RoB, moderate heterogeneity small sample size, and publication bias	Tang 2017 (33)
	MD -0.24(-0.58, 0.10); I^2^ = 74%	7 (612)	Very low	RCTs with high RoB, high heterogeneity, very small sample size, and publication bias	Liu 2017 (34)
**TG**	MD-0.23[-0.32,-0.15]; I^2^ = 13%	10 (462)	Very low	RCTs with high RoB, small sample size, and publication bias	XZ Wang 2022 (24)
	SMD -0.08[-0.32,0.16]; I^2^ = 0% TC vs. CCT MD 0.02[-0.59,0.62]; I^2^ = 86.4% TC vs. AE	5 (274) 2 (154)	Very low Very low	RCTs with high RoB, imprecision, and publication bias; RCTs with high RoB, inconsistency, imprecision, and publication bias	Cai 2022 (25)
	MD-0.99[-0.25, 0.08]; I^2^ = 66%	10 (563)	Very low	RCTs with high RoB, moderate heterogeneity, small sample size, publication bias	Yin 2022 (26)
	SMD -0.40[-0.72, -0.07]; I^2^ = 76% TC vs. CCT	9 (745)	Very low	RCTs with high RoB, high heterogeneity, small sample size, and publication bias	Guo 2021 (11)
	MD 0.04[-0.22, 0.31]; I^2^ = 71% TC vs. AE	4 (332)	Very low	RCTs with high RoB, high heterogeneity, very small sample size, and publication bias	
	SMD -0.833(-1.383, 0.283); I^2^=NA	6 (NA)	Very low	RCTs with high RoB, unclear heterogeneity, unclear sample size, and publication bias	Ge 2020 (27)
	MD -0.63, [-1.84, 0.59]; I^2^ = 97% TC vs. nonexercised MD -0.32, [-1.07, 0.43]; I^2^ = 92% TC vs. AE	2 (84) 3 (167)	Very low	RCTs with high RoB, high heterogeneity, very small sample size, and publication bias	CY Wang 2017 (32)
	MD -0.33 (-0.49,-0.17); I^2^ = 63%	6 (518)	Very Low	RCTs with high RoB, moderate heterogeneity, small sample size, and publication bias	Tang 2017 (33)
	MD -0.52 (-0.85, -0.19); I^2^ = 72%	7 (612)	Very low	RCTs with high RoB, high heterogeneity, small sample size, and publication bias	Liu 2017 (34)
**HDL**	MD 0.15[0.11,0.20]; I^2^ = 39%	7 (503)	Very low	RCTs with high RoB, moderate heterogeneity, small sample size, and publication bias	XZ Wang 2022 (24)
	SMD -0.22 [-0.08, 0.52]; I^2^ = 40.8% TC vs. CCT MD 0.03[-0.04,0.10]; I^2^ = 6.24% TC vs. AE	5 (331) 3 (262)	Very low Very low	RCTs with high RoB, inconsistency, and imprecision RCTs with high RoB, imprecision, and publication bias	Cai 2022 (25)
	SMD 0.39[0.14, 0.63]; I^2^ = 61% TC vs. CCT	9 (798)	Very low	RCTs with high RoB, moderate heterogeneity, small sample size, and publication bias	Guo 2021 (11)
	SMD 0.24[0.07, 0.41]; I^2^ = 0% TC vs. AE	5 (538)	Very low	RCTs with high RoB, small sample size, and publication bias	Tang 2017 (33)
	SMD 0.458(0.063, 0.852); I^2^ = 72.7%	4 (NA)	Very low	RCTs with high RoB, moderate heterogeneity, unclear sample size, and publication bias	Ge 2020 (27)
	MD 0.22, [-0.06, 0.50]; I^2^ = 96% TC vs. nonexercised MD 0.07, [-0.06, 0.20]; I^2^ = 65% TC vs. AE	3 (131) 4 (258)	Very low	RCTs with high RoB, moderate to high heterogeneity, very small sample size, and publication bias	CY Wang 2017 (32)
	MD 0.31 (0.14, 0.47); I^2^ = 1%	6 (566)	Very low	RCTs with high RoB, small sample size, and publication bias	Liu 2017 (34)
**LDL**	MD-0.05[-0.16,-0.07]; I^2^ = 45%	7 (403)	Very low	RCTs with high RoB, moderate heterogeneity, small sample size, and publication bias	XZ Wang 2022 (24)
	SMD -0.57 [-1.39, 0.26]; I^2^ = 86.9% TC vs. CCT MD -0.18 [-0.52,-0.15]; I^2^ = 85.6% TC vs. AE	4 (223) 2 (154)	Low Very low	RCTs with high RoB and imprecision RCTs with high RoB, high heterogeneity, and imprecision	Cai 2022 (25)
	SMD -0.79 [-1.27, 0.30]; I^2^ = 88% TC vs. CCT	9 (730)	Very low	RCTs with high RoB, high heterogeneity, small sample size, and publication bias	Guo 2021 (11)
	SMD -1.252(-2.305, -0.199); I^2^=NA	3 (NA)	Very low	RCTs with high RoB, unclear heterogeneity, unclear sample size, and publication bias	Ge 2020 (27)
	MD -0.61, [-0.72, -0.50]; I^2^ = 0% TC vs. nonexercised MD 0.21, [-0.50, 0.47]; I^2^ = 0% TC vs. AE	2 (84) 2 (63)	Very low	RCTs with high RoB, high heterogeneity, small sample size, and publication bias.	CY Wang 2017 (32)
	MD -0.32 (-0.59, -0.05); I^2^ = 79%	5 (462)	Very low	RCTs with high RoB, high heterogeneity, small sample size, and publication bias.	Liu 2017 (34)
**BMI**	MD -1.54 [-2.23, -0.85]; I^2^ = 44.9% TC vs. CCT {Yu, 2018 #1760}{项俊之, 2017 #1762}{黄苏萍, 2017 #1765}E	6 (396) 3 (246)	Very low Very low	RCTs with high RoB, high heterogeneity, and imprecision RCTs with high RoB, imprecision, and publication bias	Cai 2022 (25)
	MD-1.18[-1.80,-0.56]; I^2^ = 0%	7 (391)	Very low	RCTs with high RoB, publication bias	Yin 2022 (26)
	MD -1.15[-1.79, -0.51]; I^2^ = 0% TC vs. CCT	5 (358)	Very low	RCTs with high RoB, very small sample size, and publication bias	Guo 2021 (11)
	WMD -0.82[-1.28, -0.37]; I^2^ = 26.3%	6 (388)	Very low	RCTs with high RoB, very small sample size, and publication bias	Zhou 2019 (14)
	MD -0.62[-1.14, -0.11]; I^2^ = 44%	4 (224)	Very low	RCTs with high RoB, moderate heterogeneity, very small sample size, and publication bias.	Yu 2018 (30)
	MD -1.64 (-2.35,-0.92); I^2^ = 22%	4 (316)	Very low	RCTs with high RoB, very small sample size, and publication bias	Tang 2017 (33)
**Balance**					
**ABC**	MD 9.26 [6.68, 11.83]; I^2^ = 91%	4 (110)	Very Low	RCTs with high RoB, high heterogeneity, very small sample size, and publication bias	Wang 2022 (10)
**single-leg stance**	MD 8.38 [4.02, 12.74]; I^2^ = 45%	4 (131)	Very Low	RCTs with high RoB, moderate heterogeneity, very small sample size, and publication bias	Wang 2022 (10)
	WMD 2.72(-3.29, 8.71); I^2^ = 63.8%	3 (113)	Very Low	RCTs with high RoB, moderate heterogeneity, very small sample size, and publication bias.	Zhou 2019 (14)
**QoL**					
**Physical function (PF)**	MD 9.26 [5.14, 13.38]; I^2^ = 0%	3 (150)	Low	RCTs with high RoB and imprecision	Cai 2022 (25)
	WMD 7.07 [0.79,13.35]; I^2^ = 79.6%	5 (389)	Very Low	RCTs with high RoB, high heterogeneity, very small sample size, and publication bias.	Zhou 2019 (14)
	MD 5.54 (-0.99,12.07); I^2^ = 80%	4 (350)	Very Low	RCTs with high RoB, high heterogeneity, very small sample size, and publication bias.	Tang 2017 (33)
	MD 3.96 [-1.24, 9.17]; I^2^ = 67%	4 (347)	Very Low	RCTs with high RoB, moderate heterogeneity, very small sample size, and publication bias.	Liu 2017 (34)
**Role-physical function (RP)**	MD 11.77 [7.28, 16.26]; I^2^ = 0%	3 (150)	Low	RCTs with high RoB and imprecision	Cai 2022 (25)
	MD 8.81(4.89,12.74); I^2^ = 0%	4 (350)	Very Low	RCTs with high RoB, very small sample size, and publication bias	Tang 2017 (33)
	MD 7.24 [2.29, 12.19]; I^2^ = 38%	4 (347)	Very Low	RCTs with high RoB, low heterogeneity, very small sample size, and publication bias	Liu 2017 (34)
**Body pain (BP)**	MD 4.34 [-0.31, 8.99]; I^2^ = 0%	3 (150)	Very Low	Included RCTs with high RoB, imprecision, and publication bias	Cai 2022 (25)
	WMD 4.30 (0.83,7.77); I^2^ = 39.2%	5 (389)	Very Low	RCTs with high RoB, very small sample size, publication bias	Zhou 2019 (14)
	MD 3.96 (0.46,7.45); I^2^ = 35%	4 (350)	Very Low	RCTs with high RoB, low heterogeneity, very small sample size, and publication bias	Tang 2017 (33)
	MD 2.85 [-0.40, 6.10]; I^2^ = 45%	4 (347)	Very Low	RCTs with high RoB, moderate heterogeneity, very small sample size, publication bias	Liu 2017 (34)
**General health (GH)**	MD 8.79 (5.49,12.09); I^2^ = 49%	4 (350)	Very Low	RCTs with high RoB, moderate heterogeneity, very small sample size, publication bias	Tang 2017 (33)
**Vitality (VT)**	MD 6.34 [1.63, 11.06]; I^2^ = 0%	3 (150)	Very Low	RCTs with high RoB, imprecision, and publication bias	Cai 2022 (25)
	MD 3.60 (0.68, 6.52); I^2^ = 0%	4 (350)	Very Low	RCTs with high RoB, very small sample size, and publication bias	Tang 2017 (33)
	MD 3.54 [0.79, 6.28]; I^2^ = 0%	4 (347)	Very Low	RCTs with high RoB, very small sample size, and publication bias	Liu 2017 (34)
**Social function (SF)**	MD 11.82 [3.38, 20.26]; I^2^ = 64.4%	3 (150)	Very Low	Included RCTs with high RoB, imprecision, and publication bias	Cai 2022 (25)
	WMD 13.84 (6.22, 21.47); I^2^ = 86.0%	6 (425)	Very Low	IRCTs with high RoB, high heterogeneity, and publication bias	Zhou 2019 (14)
	MD 8.26 (2.53, 13.99); I^2^ = 68%	4 (350)	Very Low	RCTs with high RoB, moderate heterogeneity, very small sample size, and publication bias	Tang 2017 (33)
	MD 5.45 [2.46, 8.45]; I^2^ = 8%	4 (347)	Very Low	RCTs with high RoB, very small sample size, and publication bias	Liu 2017 (34)
**Role-Emotional Function (RE)**	MD 10.67 [5.64, 15.70]; I^2^ = 0%	3 (150)	Low	RCTs with high RoB and imprecision	Cai 2022 (25)
	MD 7.31 (3.89,10.730; I^2^ = 0%	4 (350)	Very Low	RCTs with high RoB, very small sample size, and publication bias	Tang 2017 (33)
	MD 6.79 [2.83, 10.76]; I^2^ = 0%	4 (347)	Very Low	RCTs with high RoB, very small sample size, and publication bias	Liu 2017 (34)
**Mental Health (MH)**	MD 7.17 [0.04, 14.29]; I^2^ = 68.7%	3 (150)	Very Low	Included RCTs with high RoB, inconsistency, and imprecision	Cai 2022 (25)
	MD 3.57(-0.67,7.81); I^2^ = 56%	4 (350)	Very Low	RCTs with high RoB, moderate heterogeneity, very small sample size, and publication bias	Tang 2017 (33)
	MD 3.83 [1.24, 6.41]; I^2^ = 47%	4 (347)	Very Low	RCTs with high RoB, moderate heterogeneity, very small sample size, and publication bias.	Liu 2017 (34)
**Total Score**	MD 45.47(18.24,72.71); I^2^ = 0%	2 (264)	Very Low	RCTs with high RoB, low heterogeneity, very small sample size, and publication bias	Tang 2017 (33)
	MD 3.67 [0.68, 6.67]; I^2^ = 17%	4 (347)	Very Low	RCTs with high RoB, low heterogeneity, very small sample size, and publication bias	Liu 2017 (34)
**QoL** **(physical domain)**	MD -5.92[-0.68, -11.16]; I^2^ = 54%	5 (226)	Very Low	RCTs with high RoB, moderate heterogeneity, very small sample size, and publication bias	Yu 2018 (30)
**QoL** **(mental domain)**	MD 6.54 [0.77,12.31]; I^2^ = 61%	5 (189)	Very Low	RCTs with high RoB, moderate heterogeneity, very small sample size, and publication bias.	Yu 2018 (30)

CCT, clinical conventional therapy; AE, aerobic exercise; HbA1c, glycated hemoglobin; BG, blood glucose; FBG, fasting blood glucose; PBG, postprandial blood glucose; FINS, fasting insulin of serum; HOMA-IR, index of homeostasis model assessment of insulin resistance; TCh, total cholesterol; TG, triglyceride; HDL &LDL, high-density lipoprotein & low-density lipoprotein; BMI, body mass index; QoL, quality of life; MD, mean difference; SMD, standard mean difference.

## 4 Discussion

### 4.1 Summary of study findings

This overview has summarized the effectiveness and safety of Tai Chi for adult patients with T2DM, based on 17 SRs of RCTs. Only 1 SR (16) drew negative conclusions, while the remaining 16 reported positive conclusions on different outcomes. Most SRs were critically low quality (15/17), with a high risk of bias (14/17), according to the AMSTAR2 and ROBIS, respectively. The CCA (12.14%) indicated a high degree of overlap, possibly suggesting redundant reviews. GRADE assessment rated all evidence as low or very low quality.

### 4.2 Effects of Tai Chi for T2DM and possible ways to probe its working mechanisms

The current study’s findings highlight Tai Chi’s effectiveness in regulating blood glucose and BMI and enhancing the Quality of Life (QoL) of Type 2 Diabetes Mellitus (T2DM) patients, particularly when compared to usual care or no treatment. However, outcomes regarding insulin resistance, blood lipids, and balance exhibit inconsistency. Notably, Tai Chi demonstrates the potential to ameliorate social function in T2DM patients. Social participation is an important component of the International Classification of Functioning, Disability, and Health (ICF) framework (40), the very basics of rehabilitation. Facilitating the diffusion of Tai Chi exercise under different backgrounds and cultural contexts serves as a way to promote socialization among T2DM patients and participation in the Tai Chi programs themselves (13).

Beyond its impact on physical health, Tai Chi’s influence on motor ability, cognition, and balance in T2DM patients warrants further exploration in future investigations. Attention to the dose-effect relationship of Tai Chi is crucial, as prolonged engagement yields greater benefits (6, 30). While two SRs (14, 36) were inconsistent on balance, Tai Chi still holds promise as a valuable intervention for improving balance, a critical concern for elderly patients, particularly those with diabetic peripheral neuropathy and a high fall risk. In alignment with recommendations by the American Diabetes Association, Tai Chi is suggested as a biweekly to triweekly exercise regimen for older adults with diabetes, specifically for enhancing flexibility and balance (41). Substantiating these claims, an SR published in 2020 (13) also supported Tai Chi’s capacity to enhance balance in T2DM patients.

While Tai Chi may not exhibit unparalleled performance compared to other forms of exercise, it presents distinct advantages in certain aspects. Recent research underscores Tai Chi’s potential as a viable cognitive treatment for older adults grappling with T2DM and mild cognitive impairment (4). Despite sharing aerobic traits with other exercises, Tai Chi’s efficacy cannot be solely attributed to its moderate intensity and energy expenditure. Rooted in traditional Chinese practice, Tai Chi combines dynamic movement and meditation, encapsulating the principles of body regulation (*Tiaoshen*), mind regulation (*Tiaoxin*), and respiration regulation (*Tiaoxi*), as elucidated in prior reviews (42, 43). Researchers are increasingly intrigued by its operational mechanisms and are undertaking mechanism-oriented investigations. Physiologically, elder Tai Chi practitioners exhibit heightened cutaneous microcirculatory function compared to sedentary controls, partly attributed to augmented nitric oxide release (44). Individuals with chronic conditions like T2DM usually have alterations in brain function (45); therefore, Tai Chi’s impact might be rooted in central nervous system modulation. Neuroimaging methods, including functional magnetic resonance imaging (fMRI) and functional near-infrared spectroscopy (fNIRS), have delved into Tai Chi’s mind-regulation potential, including cognitive impairment (46) and subthreshold depression (47). Tai Chi’s body-regulating effects have been explored via omics-based approaches like metagenomics and metabolomics, particularly in systematic ailments like metabolic syndrome, diabetes, and hypertension. Biomechanical analyses employing motion analysis systems and electromyography have investigated its influence on posture control, balance, and coordination (48). Respiratory conditions like chronic obstructive pulmonary disease have been a focal point for understanding Tai Chi’s respiratory regulation. In summary, Tai Chi operates holistically, with a multifaceted mechanism instead of singular elucidation. Given its triad of distinctive attributes, future studies should integrate diverse technologies to unravel Tai Chi’s mechanisms from multifarious perspectives.

### 4.3 Suggestions for better reporting and methodological quality of SRs and overview of Tai Chi

As commonly acknowledged, the PRISMA guideline serves as a tool for authors to enhance reporting quality and should ideally be consulted prior to or during the conduct of an SR, rather than solely as a *post-hoc* assessment tool. In this overview, five SRs (11, 16, 25, 26, 36) were reported along with the PRISMA statement. A previous analysis of Tai Chi studies (39) highlighted deficiencies in certain PRISMA items, notably encompassing protocol and registration, search strategy, risk of bias across studies, additional analysis, and funding details. Addressing these areas is imperative for bolstering the overall reporting quality of SRs.

Methodological appraisal was executed through both AMSTAR 2 and ROBIS assessments. The majority of the SRs demonstrated critically low quality (15/17) and exhibited a high risk of bias (14/17). Concerning AMSTAR 2, several SRs fell short in delineating study protocols or registrations, justifying the choice of Randomized Controlled Trials (RCTs) as the selected study design, explaining exclusions with rationales, and detailing study funding sources.

The significance of preregistration and protocol in SRs cannot be understated; they substantially diminish reporting bias, enhance study transparency, curtail redundant publication, and economize authors’ time and effort. Emphasizing formal and standardized preregistration and protocol for SRs contributes to their reliability and should be actively advocated and adopted by SR authors. To ensure coherence and consistency, this overview specifically focused on SRs based solely on RCTs, thereby mitigating the heterogeneity stemming from divergent study types. Additionally, it is crucial for funding sources and potential conflicts of interest to be unequivocally disclosed, thereby augmenting confidence in both RCTs and SRs.

For the ROBIS results, deficiencies predominantly emerged in the identification and selection of studies (Domain 2), collection and study appraisal (Domain 3), and synthesis and findings (Domain 4), largely encompassing methodological aspects. It is worth noting that many SRs included in this overview employed the original version of the RoB tool, rather than the updated RoB 2. While RoB 2 accommodates a broader spectrum of study types, including cluster RCTs and crossover RCTs, it entails a more intricate evaluation process. Consequently, if an SR encompasses study types beyond parallel RCTs, the adoption of RoB 2 is recommended.

SRs have recently gained considerable popularity in recent years, yet the quality of many published SRs falls below optimal standards, with methodology being a prevalent contributing factor. Enhancing reporting and methodology quality necessitates the integration of tools such as PRISMA, as well as assessment instruments like AMSTAR 2 and ROBIS. Raising awareness among SR authors regarding the pivotal role of refining methodology and reporting quality is fundamental to ensuring robust evidence generation from study findings.

Safety concerns related to exercise therapy for Tai Chi have been inadequately reported in many SRs. Given the potential for harm from sports injuries during exercise, heightened attention is warranted toward AEs associated with Tai Chi interventions. Regrettably, only four SRs (11, 16, 25, 31) reported AEs related to Tai Chi exercise therapy for T2DM. To comprehensively evaluate the safety profile of Tai Chi interventions, an increased number of studies, with meticulous AE reporting, is indispensable.

Additionally, due to the nature of Tai Chi exercise, designing and conducting a placebo-controlled RCT poses considerable challenges, particularly in locales where traditional exercises like Tai Chi are prevalent, as in China. Consequently, numerous RCTs on Tai Chi suffer from a heightened risk of bias, particularly concerning blinding, sequence generation, and allocation concealment. For future refinement, specialized guidelines and tools tailored to RCTs on Tai Chi could be pivotal in elevating study quality. In the future, guidelines and tools tailored for RCTs on Tai Chi may be necessary to improve study quality. Drawing inspiration from the Standards for Reporting Interventions in Clinical Trials of Acupuncture (STRICTA) for RCTs on acupuncture could serve as a useful model for developing such resources.

Overviews have been categorized as SRs, and the previous reporting checklist for SRs like PRISMA is also applicable to overviews. However, existing standards have not adequately addressed the issue of overlapping content, despite the potential indication of excessive duplication of SRs (22). This concern has been specifically tackled in the PRIO-harms checklist (17), which incorporates a dedicated section for assessing overlap within this context. To quantify the extent of overlap, the CCA metric has been proposed as a suitable method for evaluating overlap in overviews. The utility of the PRIO-harms checklist has been demonstrated in various studies. It has been successfully applied to overviews of acupuncture and moxibustion (49), traditional Chinese medicine for ulcerative colitis (50), and the application of artificial intelligence for prognostics and health management (51). For the enhancement of reporting standards in overviews or umbrella reviews, we strongly advocate the adoption of the PRIO-harms checklist.

### 4.4 Strengths and limitations

This comprehensive overview presents the most up-to-date evidence regarding the use of Tai Chi for T2DM, providing a reference for clinical practice. Furthermore, the pilot version of the PRIO-harms checklist, which incorporates assessments of overlapping content, was employed to enhance the reporting quality of this overview. Derived in 2018 from the PRISMA, PRISMA harms, and PRISMA-P guidelines, this checklist aims to ensure balanced reporting of both benefits and harms in overviews.

Nevertheless, certain limitations exist within this overview. Firstly, the inclusion criteria were confined to RCT-based SRs, thereby excluding other types of literature. To expand the scope of analysis, it is advisable to consider incorporating various study designs related to Tai Chi for T2DM in future aggregated analyses. Secondly, the studied population exclusively encompassed adult patients diagnosed with T2DM, potentially neglecting other age groups such as children and teenagers in forthcoming research endeavors. Additionally, a notable proportion of the included SRs exhibited suboptimal methodological quality, displaying a high susceptibility to bias. Consequently, the overall quality of evidence, as evaluated by the GRADE framework, was classified as low or very low, thereby restraining the confidence level of the overview’s concluding remarks. Lastly, while the inclusion of the PRIO-harms checklist represents a notable aspect of this study, it is imperative to acknowledge that the checklist is currently in a pilot phase. The validation of this checklist through rigorous assessments across diverse SRs spanning various subject areas remains relatively limited. Furthermore, its dissemination through established platforms, such as the EQUATOR Network (https://www.equator-network.org/), has been somewhat constrained, which in turn could potentially hinder its broader accessibility to the public.

## 5 Conclusions

Tai Chi shows promise as a potentially effective and safe lifestyle intervention for adults with T2DM, particularly in improving HbA1c, FBG, BMI, and overall quality of life (QoL). However, these results should be cautiously interpreted due to methodological flaws observed in the current SRs and the low quality of the SRs based on GRADE. Furthermore, there is a compelling need for additional well-designed, high-quality RCTs and SRs to establish robust and conclusive evidence regarding the efficacy of Tai Chi for managing T2DM in the future.

## Data availability statement

The original contributions presented in the study are included in the article/**Supplementary Material**. Further inquiries can be directed to the corresponding authors.

## Author contributions

FRZ, XC, and XL contributed equally to this manuscript. Study conceptualization: RJ and FRZ. Literature search, and data collection and analysis: FRZ, XC, XL, XS, and TL. Manuscript preparation: FRZ, XC, and XL. Manuscript revision: FZ and RJ. All authors contributed to the article and approved the submitted version.
