# Supplementary material for: Lifestyle intervention Tai Chi for adult patients with type 2 diabetes mellitus: a PRIO-harms based overview of 17 systematic reviews
Source: Front Endocrinol (Lausanne). 2024 Jan 17;14:1208202. doi: 10.3389/fendo.2023.1208202 (PMC10829778; doi:10.3389/fendo.2023.1208202)
Supplement: Supplementary file 1 [file Table_1.docx]

**Table 1 GRADE evidence profile of included SRs**

| **SRs** | **Outcomes** | **Certainty assessment** | | | | | |  | **No. of patients** | | **Relative effect (95%CI);**  **heterogeneity** | **Model** | **Quality** |
| --- | --- | --- | --- | --- | --- | --- | --- | --- | --- | --- | --- | --- | --- |
|  |  | **No. of RCTs** | **Limitations** | **Inconsistency** | **Indirectness** | **Imprecision** | **Publication bias** |  | **TC** | **control** |  |  |  |
| **XZ Wang2022** | **HbA1c** | 16 | -2 | -2 | 0 | 0 | 0 |  | 494 | 497 | MD -1.10[-1.78, -0.24]; I^2^=98% | random | ⊕OOO Very low |
|  | **FBG** | 18 | -2 | -2 | 0 | 0 | 0 |  | 542 | 527 | MD-0.79[-1.12,-0.64]; I^2^=75% | random | ⊕OOO Very low |
|  | **TCh** | 10 | -2 | -2 | 0 | 0 | +1 |  | 338 | 324 | MD-0.27[-0.60,0.05]; I^2^=82% | random | ⊕OOO Very low |
|  | **TG** | 10 | -2 | 0 | 0 | 0 | +1 |  | 238 | 224 | MD-0.23[-0.32,-0.15]; I^2^=13% | fixed | ⊕OOO Very low |
|  | **HDL** | 7 | -2 | 0 | 0 | 0 | +1 |  | 254 | 249 | MD 0.15[0.11,0.20]; I^2^=39% | fixed | ⊕OOO Very low |
|  | **LDL** | 7 | -2 | -2 | 0 | -1 | +1 |  | 204 | 199 | MD-0.05[-0.16,-0.07]; I^2^=45% | fixed | ⊕OOO Very low |
| **Cai2022** | The quality of the evidence of the outcomes was calculated using the GRADE approach , all of which was found to be " low " or " very low quality. Compared with the usual care, the evidence quality of the effects of Tai Chi on HbA1c, FBG, IR, FIN, TC, TG, HDL,LDL, blood pressure, BMI, and QoL was low or very low (Table 6);while compared with other exercise, the evidence quality for Tai Chi's impact on HbA1c, FBG, TC, TG, HDL, LDL, BMI, and waist circumference was low or very low. | | | | | | | | | | | | |
| **Yin2022** | **HbA1c** | 8 | -2 | -2 | 0 | 0 | +1 |  | 254 | 245 | MD -0.81[-1.31,-0.32]; I^2^=85% | random | ⊕OOO Very low |
|  | **FBG**  TC vs. CCT  TC vs. AE | 9  5 | -2  -2 | -2  0 | 0  0 | -1  -1 | +1  +1 |  | 194  154 | 191  190 | MD -0.85[-1.22,-0.38] ; I^2^=75%  MD 0.14[-0.32, 0.59] ; I^2^=39% | random  random | ⊕OOO Very low  ⊕OOO Very low |
|  | **FINS** | 6 | -2 | -2 | 0 | -1 | +1 |  | 142 | 138 | MD-3.00[-4.04,-1.97] ; I^2^=67% | fixed | ⊕OOO Very low |
|  | **TCh** | 10 | -2 | -1 | 0 | 0 | +1 |  | 260 | 239 | MD-0.11[-0.32,0.09]; I^2^=46% | random | ⊕OOO Very low |
|  | **TG** | 10 | -2 | -1 | 0 | 0 | +1 |  | 292 | 271 | MD-0.99[-0.25, 0.08] ; I^2^=66% | random | ⊕OOO Very low |
|  | **BMI** | 7 | -2 | 0 | 0 | -1 | +1 |  | 208 | 183 | MD-1.18[-1.80,-0.56] ; I^2^=0% | fixed | ⊕OOO Very low |
| **Wang2022** | **Glucose** | 5 | -1 | 0 | 0 | -1 | +1 |  | 138 | 137 | MD -12.47 [-21.20,-3.73]; I^2^=32% | fixed | ⊕OOO Very low |
|  | **ABC scale** | 4 | 0 | -2 | 0 | -2 | +1 |  | 55 | 55 | MD 9.26 [6.68, 11.83]; I^2^=91% | random | ⊕OOO Very low |
|  | **Single limb standing test** | 4 | -1 | -1 | 0 | -2 | +1 |  | 66 | 65 | MD 8.38 [4.02, 12.74]; I^2^=45% | fixed | ⊕OOO Very low |
| **Guo 2021** | **HbA1c1** |  |  |  |  |  |  |  |  |  |  |  |  |
|  | TC vs. CCT | 9 | -2 | -2 | 0 | 0 | +1 |  | 377 | 372 | MD -0.73[-1.03, -0.43]; I^2^=82% | random | ⊕OOO Very low |
|  | TC vs. AE | 5 | -2 | -1 | 0 | 0 | +1 |  | 255 | 249 | MD -0.33[-0.61, -0.04]; I^2^=48% | random | ⊕OOO Very low |
|  | **FBG** |  |  |  |  |  |  |  |  |  |  |  |  |
|  | TC vs. CCT | 15 | -1 | -1 | 0 | 0 | +1 |  | 519 | 504 | SMD -0.62[-0.85, -0.40]; I^2^=63% | random | ⊕OOO Very low |
|  | TC vs. AE | 8 | -2 | -1 | 0 | 0 | +1 |  | 312 | 307 | SMD -0.03[-0.30, 0.23]; I^2^=60% | random | ⊕OOO Very low |
|  | **FINs** TC vs. CCT | 3 | -2 | 0 | 0 | -1 | +1 |  | 128 | 127 | MD -1.02[-1.39, -0.64]; I^2^=0% | fixed | ⊕OOO Very low |
|  | **HOMA-IR** TC vs. CCT | 7 | -2 | -1 | 0 | 0 | +1 |  | 252 | 248 | SMD -0.65 [-1.01, -0.30]; I^2^=68% | random | ⊕OOO Very low |
|  | **TCh** |  |  |  |  |  |  |  |  |  |  |  |  |
|  | TC vs. CCT | 11 | -2 | -2 | 0 | 0 | +1 |  | 442 | 426 | SMD -0.51[-0.88, -0.14]; I^2^=84% | random | ⊕OOO Very low |
|  | TC vs. AE | 5 | -2 | 0 | 0 | 0 | +1 |  | 213 | 210 | MD -0.08[-0.24, 0.09]; I^2^=36% | fixed | ⊕OOO Very low |
|  | **TG** |  |  |  |  |  |  |  |  |  |  |  |  |
|  | TC vs. CCT | 9 | -2 | -1 | 0 | 0 | +1 |  | 381 | 364 | SMD -0.40[-0.72, -0.07]; I^2^=76% | random | ⊕OOO Very low |
|  | TC vs. AE | 4 | -2 | -1 | 0 | -1 | +1 |  | 170 | 162 | MD 0.04[-0.22, 0.31]; I^2^=71% | random | ⊕OOO Very low |
|  | **HDL** |  |  |  |  |  |  |  |  |  |  |  |  |
|  | TC vs. CCT | 9 | -2 | -1 | 0 | 0 | +1 |  | 406 | 392 | SMD 0.39[0.14, 0.63]; I^2^=61% | random | ⊕OOO Very low |
|  | TC vs. AE | 5 | -2 | 0 | 0 | 0 | +1 |  | 268 | 270 | SMD 0.24[0.07, 0.41]; I^2^=0% | fixed | ⊕OOO Very low |
|  | **LDL** TC vs. CCT | 9 | -2 | -2 | 0 | 0 | +1 |  | 372 | 358 | SMD -0.79 [-1.27, 0.30]; I^2^=88% | random | ⊕OOO Very low |
|  | **BMI** TC vs. CCT | 5 | -2 | 0 | 0 | -1 | +1 |  | 185 | 173 | MD -1.15[-1.79, -0.51]; I^2^=0% | fixed | ⊕OOO Very low |
| **Ge 2020** | **HbA1c** | 7 | -2 | -2 | 0 | -2 | +1 |  | NA | NA | SMD -0.585(-0.784,−0.386);I^2^=95.2% | random | ⊕OOO Very low |
|  | **FBG** | 9 | -2 | -1 | 0 | -2 | +1 |  | NA | NA | SMD -0.607(-0.930,−0.284);I^2^=68.2% | random | ⊕OOO Very low |
|  | **TCh** | 7 | -2 | -2 | 0 | -2 | +1 |  | NA | NA | SMD -0.418(-0.897,0.061); I^2^=84.9% | random | ⊕OOO Very low |
|  | **TG** | 6 | -2 | -2 | 0 | -2 | +1 |  | NA | NA | SMD -0.833(-1.383, 0.283); I^2^:NA | random | ⊕OOO Very low |
|  | **HDL** | 4 | -2 | -1 | 0 | -2 | +1 |  | NA | NA | SMD 0.458(0.063, 0.852); I^2^=72.7% | random | ⊕OOO Very low |
|  | **LDL** | 3 | -2 | -2 | 0 | -2 | +1 |  | NA | NA | SMD -1.252(-2.305, -0.199); I^2^=NA | random | ⊕OOO Very low |
| **Xun 2019** | **FBG** | 39 | -2 | 0 | 0 | 0 | 0 |  | 1044 | | SMD -0.38(-0.46, -0.29); I^2^=42.3% | random | ⊕OOO Very low |
|  | **HbA1c** | 24 | -2 | -1 | 0 | 0 | 0 |  | 774 | | SMD -0.50(-0.60, -0.40); I^2^=74.4% | random | ⊕OOO Very low |
|  | **FINs** | 12 | -2 | 0 | 0 | -1 | 0 |  | 308 | | SMD -0.28(-0.44, -0.12); I^2^=34.5% | random | ⊕OOO Very low |
| **Su2019** | **HbA1c** | 7 | -2 | -2 | 0 | 0 | +1 |  | 255 | 260 | MD -0.76% [-1.38, -0.14]; I^2^=97% | random | ⊕OOO Very low |
|  | **FBG** | 14 | -2 | -2 | 0 | 0 | +1 |  | 1128 | 1102 | SMD -0.85 [-1.17, -0.52]; I^2^=83% | random | ⊕OOO Very low |
|  | **2hPBG** | 2 | -2 | 0 | 0 | -2 | +1 |  | 70 | 70 | MD -1.03 [-1.34, -0.73]; I^2^=0% | fixed | ⊕OOO Very low |
|  | **HOMA-IR** | 4 | -2 | -1 | 0 | -1 | +1 |  | 190 | 187 | MD -0.69[-0.06, -1.31]; I^2^=59% | random | ⊕OOO Very low |
|  | **TCh** | 5 | -2 | -2 | 0 | 0 | +1 |  | 602 | 636 | SMD -0.67[-1.38, 0.03]; I^2^=92% | random | ⊕OOO Very low |
| **Zhou2019** | **HbA1c** | 12 | -1 | -1 | 0 | 0 | +1 |  | 388 | 326 | WMD -0.53(-0.62, -0.44); I^2^=43.4% | random | ⊕OOO Very low |
|  | **FBG** | 21 | -1 | -1 | 0 | 0 | +1 |  | 601 | 514 | SMD -0.67(-0.87, -0.47); I^2^=53.2% | random | ⊕OOO Very low |
|  | **FINS** | 8 | 0 | -2 | 0 | 0 | +1 |  | 257 | 243 | SMD -0.32[0.71,0.07]; I^2^=73.3% | random | ⊕OOO Very low |
|  | **HOMA-IR** | 5 | 0 | 0 | 0 | -1 | +1 |  | 175 | 157 | WMD -0.41 [-0.78, -0.04]; I^2^=0.0% | fixed | ⊕OOO Very low |
|  | **TCh** | 9 | -1 | -1 | 0 | 0 | +1 |  | 347 | 311 | SMD-0.59[-0.90,-0.27]; I^2^=66.6% | random | ⊕OOO Very low |
|  | **BMI** | 6 | -1 | 0 | 0 | -1 | +1 |  | 201 | 187 | WMD-0.82(-1.28, -0.37); I^2^=26.3% | fixed | ⊕OOO Very low |
|  | **SBP** | 5 | 0 | -1 | 0 | -1 | +1 |  | 151 | 139 | WMD -10.03[-15.78, -4.29];I^2^=55.4% | random | ⊕OOO Very low |
|  | **DBP** | 5 | 0 | 0 | 0 | -1 | +1 |  | 151 | 139 | WMD -4.85[-8.23, -1.47]; I^2^=33.1% | random | ⊕OOO Very low |
|  | **Balance** | 3 | -1 | -1 | 0 | -2 | +1 |  | 54 | 59 | WMD 2.72(-3.29, 8.71); I^2^=63.8% | random | ⊕OOO Very Low |
|  | **Physical function** | 4 | -1 | -2 | 0 | -1 | +1 |  | 198 | 191 | WMD 7.07[0.79, -13.35]; I^2^=79.6% | random | ⊕OOO Very Low |
|  | **Bodily pain** | 4 | -1 | 0 | 0 | -1 | +1 |  | 198 | 191 | WMD 4.30(0.83, 7.77); I^2^=39.2% | random | ⊕OOO Very Low |
|  | **Social function** | 5 | -1 | -2 | 0 | -1 | +1 |  | 215 | 211 | MD 13.84(6.22, 21.47); I^2^=86.0% | random | ⊕OOO Very Low |
| **Yu 2018** | **HbA1c** | 5 | -1 | -2 | 0 | -1 | 0 |  | 154 | 140 | MD -1.25[-−2.53, 0.03]; I^2^=99% | random | ⊕OOO Very Low |
|  | **FBG** | 6 | -1 | -1 | 0 | -1 | +1 |  | 157 | 146 | MD -1.14[−1.78, −0.50]; I^2^=67% | random | ⊕OOO Very Low |
|  | **BMI** | 4 | -1 | -1 | 0 | -1 | +1 |  | 117 | 107 | MD -0.62[−1.14, −0.11]; I^2^=44% | fixed | ⊕OOO Very Low |
|  | **QoL-physical domain** | 5 | -1 | -1 | 0 | -1 | +1 |  | 115 | 111 | MD -5.92[−0.68, −11.16]; I^2^=54% | random | ⊕OOO Very Low |
|  | **QoL-mental domain** | 5 | -1 | -1 | 0 | -2 | +1 |  | 98 | 91 | MD 6.54 [0.77,12.31]; I^2^=61% | random | ⊕OOO Very Low |
| **Chao2018** | **HbA1c**  TC vs. nonexercise  TC vs. AE | 7  7 | -2  -2 | -1  -1 | 0  0 | -1  -1 | +1  +1 |  | 147  180 | 146  192 | MD -0.73[-0.95, -0.52]; I^2^=57%  MD -0.19[-0.37, 0.00]; I^2^=65% | random  random | ⊕OOO Very Low  ⊕OOO Very Low |
|  | **FBG**  TC vs. nonexercise  TC vs. AE | 10  7 | -2  -2 | -2  -1 | 0  0 | 0  -1 | +1  +1 |  | 245  173 | 244  169 | MD -1.39[-1.95, -0.84]; I^2^=86%  MD -0.21[-0.61,0.19]; I^2^=63% | random  random | ⊕OOO Very Low  ⊕OOO Very Low |
|  | **2hPBG**  TC vs. nonexercise  TC vs. AE | 5  3 | -2  -2 | 0  -1 | 0  0 | -2  -2 | +1  +1 |  | 82  42 | 80  42 | MD -2.07 [-2.89, -1.26]; I^2^=0%  MD -0.44[-1.42, 0.54]; I^2^=0% | random  random | ⊕OOO Very Low  ⊕OOO Very Low |
| **Q Wang2017** | **HbA1c** | 8 | -2 | -2 | 0 | 0 | +1 |  | 285 | 298 | SMD-0.68[-0.86, -0.51]; I^2^=88% | random | ⊕OOO Very Low |
|  | **FBG** | 12 | -2 | 0 | 0 | 0 | +1 |  | 347 | 361 | SMD -0.24[-0.39, -0.09]; I^2^=38.3% | random | ⊕OOO Very Low |
| **CY Wang2017** | **HbAlc**  TC vs. nonexercised  TC vs. AE | 8  5 | -1  -1 | -2  -1 | 0  0 | -1  -1 | +1  +1 |  | 180  151 | 176  152 | MD -0.98 [-1.65, -0.31]; I^2^=97%  MD -0.25 [-0.52, 0.01]; I^2^=68% | random  random | ⊕OOO Very Low  ⊕OOO Very Low |
|  | **FBG**  TC vs. nonexercised  TC vs. AE | 9  5 | -2  -2 | -2  -1 | 0  0 | 0  -1 | +1  +1 |  | 242  144 | 238  141 | MD -1.11 [-1.65, -0.56]; I^2^=79%  MD -0.32 [-0.80, 0.17]; I^2^=64% | random  random | ⊕OOO Very Low  ⊕OOO Very Low |
|  | **2hPBG**  TC vs. nonexercised  TC vs. AE | 3  1 | -2  -2 | 0  0 | 0  0 | -2  -2 | +1  +1 |  | 53  13 | 52  14 | MD -2.05 [-3.12, -0.09]; I^2^=0%  MD -0.67 [-2.17, 0.83]; NA | fixed  random | ⊕OOO Very Low  ⊕OOO Very Low |
|  | **TCh**  TC vs. nonexercised  TC vs. AE | 4  4 | -2  -2 | -2  -1 | 0  0 | -1  -1 | +1  +1 |  | 111  131 | 104  127 | MD -0.71, [-1.15, -0.27]; I^2^=83%  MD -0.04, [-0.33, 0.25]; I^2^=68% | random  random | ⊕OOO Very Low  ⊕OOO Very Low |
|  | **TG**  TC vs. nonexercised  TC vs. AE | 2  3 | -2  -2 | -2  -2 | 0  0 | -2  -2 | +1  +1 |  | 44  88 | 40  79 | MD -0.63, [-1.84, 0.59]; I^2^=97%  MD -0.32, [-1.07, 0.43]; I^2^=92% | random  random | ⊕OOO Very Low  ⊕OOO Very Low |
|  | **HDL**  TC vs. nonexercised  TC vs. AE | 3  4 | -2  -2 | -2  -2 | 0  0 | -2  -1 | +1  +1 |  | 87  131 | 44  127 | MD 0.22, [-0.06, 0.50]; I^2^=96%  MD 0.07, [-0.06, 0.20]; I^2^=65% | random  random | ⊕OOO Very Low  ⊕OOO Very Low |
|  | **LDL**  TC vs. nonexercised  TC vs. AE | 2  2 | -2  -2 | 0  0 | 0  0 | -2  -2 | +1  +1 |  | 44  38 | 40  25 | MD -0.61, [-0.72, -0.50]; I^2^=0%  MD 0.21, [-0.50, 0.47]; I^2^=0% | fixed  fixed | ⊕OOO Very Low  ⊕OOO Very Low |
| **Tang 2017** | **HbAlc** | 7 | -2 | -1 | 0 | 0 | +1 |  | 292 | 280 | MD -1.12[-1.87,-0.38]; I^2^=67.5% | random | ⊕OOO Very Low |
|  | **Glucose** | 7 | -2 | -1 | 0 | -1 | +1 |  | 184 | 170 | MD -0.74 [-1.32, -0.16]; I^2^=70% | random | ⊕OOO Very Low |
|  | **TCh** | 6 | -2 | -2 | 0 | 0 | +1 |  | 266 | 252 | MD 0.08 [-0.33, 0.48]; I^2^=80% | random | ⊕OOO Very Low |
|  | **TG** | 6 | -2 | -1 | 0 | 0 | +1 |  | 266 | 252 | MD -0.33 [-0.49,-0.17]; I^2^=63% | random | ⊕OOO Very Low |
|  | **BMI** | 4 | -2 | 0 | 0 | -1 | +1 |  | 164 | 152 | MD -1.64 [-2.35,-0.92]; I^2^=22% | fixed | ⊕OOO Very Low |
|  | **QoL** | 2 | -2 | 0 | 0 | -1 | +1 |  | 264 | | MD 45.47(18.24,72.71); I^2^=0% | fixed | ⊕OOO Very Low |
| **Liu 2017** | **HbAlc** | 7 | -2 | -1 | 0 | 0 | +1 |  | 327 | 318 | MD -0.59 (-0.73, -0.44); I^2^=45% | fixed | ⊕OOO Very Low |
|  | **FBG** | 9 | -2 | -1 | 0 | 0 | +1 |  | 369 | 358 | MD -0.39 (-0.54, -0.24); I^2^=47% | fixed | ⊕OOO Very Low |
|  | **TCh** | 7 | -2 | -2 | 0 | 0 | +1 |  | 312 | 300 | MD -0.24(-0.58, 0.10); I^2^=74% | random | ⊕OOO Very Low |
|  | **TG** | 7 | -2 | -2 | 0 | 0 | +1 |  | 312 | 300 | MD -0.52 (-0.85 ,-0.19); I^2^=72% | random | ⊕OOO Very Low |
|  | **HDL** | 6 | -2 | 0 | 0 | 0 | +1 |  | 288 | 278 | MD 0.31 (0.14, 0.47); I^2^=1% | fixed | ⊕OOO Very Low |
|  | **LDL** | 5 | -2 | -2 | 0 | 0 | +1 |  | 232 | 230 | MD -0.32 (-0.59, -0.05); I^2^=79% | random | ⊕OOO Very Low |
| **Zhang 2016** | **Glucose** | 4 | -2 | 0 | -2 | -2 | +1 |  | 59 | 59 | MD =-0.43[-0.03, -0.84]; I^2^=0% | fixed | ⊕OOO Very Low |
| **Lee 2014** | **HbAlc** |  |  |  |  |  |  |  |  |  |  |  |  |
|  | TC vs. AE | 2 | -2 | -1 | 0 | -2 | +1 |  | 78 | 70 | MD 0.00[-0.31, 0.31]; I^2^=0% | random | ⊕OOO Very Low |
|  | TC vs. drug  TC vs. no treatment | 3  2 | -2  -2 | -1  -2 | 0  0 | -2  -2 | +1  +1 |  | 65  44 | 62  40 | MD -0.54[-1.23, 0.15]; I^2^=14%  MD -1.58[-3.83, 0.67]; I^2^=95% | random  random | ⊕OOO Very Low  ⊕OOO Very Low |
|  | **FBG** |  |  |  |  |  |  |  |  |  |  |  |  |
|  | TC vs. AE | 4 | -2 | 0 | 0 | -1 | +1 |  | 114 | 98 | MD =-0.03[-0.49, 0.42];I^2^=39% | random | ⊕OOO Very Low |
|  | TC vs. drug | 4 | -2 | 0 | 0 | -2 | +1 |  | 95 | 93 | MD =-1.57[-2.34, -0.80];I^2^=0% | random | ⊕OOO Very Low |

Notes: **CCT:** clinical conventional therapy; **AE:** aerobic exercise; **HbA1c:** glycated hemoglobin; **BG:** blood glucose; **FBG:** fasting blood glucose; **PBG:** postprandial blood glucose; F**INS:** fasting serum insulin; **HOMA-IR:** index of homeostasis model assessment of insulin resistance; **TCh**: total cholesterol; **TG:** triglyceride; **HDL &LDL:** high-density lipoprotein & low-density lipoprotein; **BMI:** body mass index; **QoL:** quality of life; **MD:** mean difference; **SMD:** standard mean difference

**0:**no; **-1**:serious; **-2**:very serious, for publication bias, **+1** refers to “strongly suspected”, while **0** refers to “undetected”.
